# Supplementary material for: Comparative efficacy of antibiotic(s) alone or in combination of corticosteroids in adults with acute bacterial meningitis: A systematic review and network meta-analysis
Source: PLoS One. 2020 May 29;15(5):e0232947. doi: 10.1371/journal.pone.0232947 (PMC7259733; doi:10.1371/journal.pone.0232947)
Supplement: S1 Appendix — (DOCX) [file pone.0232947.s002.docx]

**Supplementary appendix**

**Comparative efficacy of different pharmacotherapies for adults with acute bacterial meningitis: a systematic review and GRADE analysis**

Ajaree Rayanakorn, Hooi-Leng Ser, Priyia Pusparajah, Kok-Gan Chan, Bey Hing Goh, Tahir Mehmood Khan, Surasak Saokaew, Shaun Lee Wen Huey, Learn Han Lee

March, 2020

**Online Supplementary Content**

[Appendix 1 The search strings used 4](#_Toc461527261)

[eFigure 1.1 Flow diagram of search strategy and study selection. 6](#_Toc461527263)

[eTable 1.1 Details of 30 studies excluded after full-text review. 7](#_Toc461527265)

[Appendix 2 Detail of treatments 9](#_Toc461527264)

[eTable 2.1 Detail of antibiotic regimens. 9](#_Toc461527265)

[Appendix 3 Characteristics of included studies 10](#_Toc461527266)

[eTable 3.1 Key characteristics of included studies. 10](#_Toc461527267)

[eTable 3.2 Description of study participants…………………………………………………………...…18](#_Toc461527267)

[Appendix 4 Risk of bias summary....………………………………………………………………………...19](#_Toc461527268)

[eFigure 4.1 Summarized risk of bias of included randomized controlled trials (RoB2.0)……………19](#_Toc461527269)

[Appendix 5 Network plot of all treatment comparisons…………………………………………………….20](#_Toc461527271)

[eFigure 5.1 All-cause mortality…………………………………………………………………………….20](#_Toc461527272)

[eFigure 5.2 Neurological sequelae………………………………………………………………………..21](#_Toc461527273)

[eFigure 5.3 Any hearing loss……………………………………………………………………………….22](#_Toc461527274)

[Appendix 6 Assessment of inconsistency for each outcome...……………………………………………23](#_Toc461527276)

[eFigure 6.1 Assessment of global inconsistency in networks using the ‘design-by-treatment’ interaction model.](#_Toc461527277) 23

[eTable 6.1 Assessment of global inconsistency in networks using the ‘design-by-treatment’ interaction model.](#_Toc461527278) 25

[eTable 6.2 Inconsistency test between direct and indirect comparisons in mixed treatment comparison for all-cause mortality](#_Toc461527278) 25

[eTable 6.3 Inconsistency test between direct and indirect comparisons in mixed treatment comparison for neurological sequelae](#_Toc461527278) 26

[eTable 6.4 Inconsistency test between direct and indirect comparisons in mixed treatment comparison for any hearing loss](#_Toc461527278) 26

[Appendix 7 Results of meta-analysis for direct comparisons](#_Toc461527279) 27

[eFigure 7.1 Results of pairwise meta-analyses for all-cause mortality](#_Toc461527280) 27

[eTable 7.1 Results of pairwise meta-analyses for all-cause mortality](#_Toc461527280) 27

[eFigure 7.2 Results of pairwise meta-analyses for neurological sequelae](#_Toc461527280) 28

[eTable 7.2 Results of pairwise meta-analyses for neurological sequelae](#_Toc461527280) 28

[eFigure 7.3 Results of pairwise meta-analyses for any hearing loss](#_Toc461527280) 29

[eTable 7.3 Results of pairwise meta-analyses for any hearing loss](#_Toc461527280) 29

[eFigure 7.4 Results of of network meta-analysis of treatment options](#_Toc461527280) 30

[eFigure 7.5 Contribution plots of treatment options](#_Toc461527280) 32

[Appendix 8 Results of network meta-analysis](#_Toc461527281) 34

[eFigure8.1 Network estimated ratios (95%CI) for all-cause mortality](#_Toc461527282) 34

[eFigure8.2 Network estimated ratios (95%CI) for neurological sequelae](#_Toc461527282) 34

[eFigure8.3 Network estimated ratios (95%CI) for any hearing loss](#_Toc461527282) 34

[Appendix 9 Treatment ranking and and surface under the cumulative ranking curves (SUCRA)](#_Toc461527288) 35

[eFigure 9.1 SUCRA ranking curves for all-cause mortality.](#_Toc461527289) 35

[eFigure 9.2 SUCRA ranking curves for neurological sequelae](#_Toc461527290) 36

[eFigure 9.3 SUCRA ranking curves for hearing loss](#_Toc461527291) 37

[Appendix 10 Evaluation of the quality of evidence using GRADE framework for primary outcomes](#_Toc461527303) 38

[eTable 10.1 Direct estimates and GRADE quality assessment of direct evidence for each pairwise comparison for primary outcomes.](#_Toc461527304) 38

e[Table 10.2 Network estimates and GRADE quality assessment for primary outcomes.](#_Toc461527305) 40

[Appendix 11 Comparison-adjusted funnel plot](#_Toc461527303) 42

[eFigure 11.1 Comparison-adjusted funnel plot for the network of all-cause mortality in all comparisons.](#_Toc461527304) 42

[eFigure 11.2 Comparison-adjusted funnel plot for the network of neurological sequelae in all comparisons.](#_Toc461527305) 43

[eFigure 11.3 Comparison-adjusted funnel plot for the network of any hearing loss in all comparisons.](#_Toc461527306) 44

[Appendix 12 Trim-and-fill method for the pair-wise meta-analysis](#_Toc461527303) 45

[eFigure 12.1 Trim-and-fill method for the pair-wise meta-analysis of all-cause mortality](#_Toc461527304) 45

[eTable 12.1 Results from the trim-and-fill method for publication bias in 6 studies for all-cause mortality](#_Toc461527304) 45

[eFigure 12.2 Trim-and-fill method for the pair-wise meta-analysis of neurological sequelae](#_Toc461527305) 46

[eTable 12.2 Results from the trim-and-fill method for publication bias in 5 studies for neurological sequelae](#_Toc461527304) 46

[eFigure 12.3 Trim-and-fill method for the pair-wise meta-analysis of any hearing loss](#_Toc461527306) 47

[eTable 12.3 Results from the trim-and-fill method for publication bias in 6 studies for any hearing loss](#_Toc461527304) 47

[Appendix 13 References](#_Toc461527303) 48

**Appendix 1: The search strings used**

**Medline (Ovid)**

bacterial meningitis.mp. or Meningitis, Bacterial/ OR (bacterial meningitis and treatment).mp. [mp=title, abstract, original title, name of substance word, subject heading word, keyword heading word, protocol supplementary concept word, rare disease supplementary concept word, unique identifier, synonyms], limit to (full text and humans and "all adult (19 plus years)")

**PubMed**

(("meningitis, bacterial"[MeSH Terms] OR ("meningitis"[All Fields] AND "bacterial"[All Fields]) OR "bacterial meningitis"[All Fields] OR ("bacterial"[All Fields] AND "meningitis"[All Fields])) OR ("meningitis, bacterial"[MeSH Terms] OR ("meningitis"[All Fields] AND "bacterial"[All Fields]) OR "bacterial meningitis"[All Fields] OR ("bacterial"[All Fields] AND "meningitis"[All Fields]))) AND ("therapy"[Subheading] OR "therapy"[All Fields] OR "treatment"[All Fields] OR "therapeutics"[MeSH Terms] OR "therapeutics"[All Fields]) AND ("loattrfull text"[sb] AND "humans"[MeSH Terms] AND "adult"[MeSH Terms])

**ScienceDirect**

(bacterial meningitis) or ("bacterial meningitis" AND "treatment") AND LIMIT-TO(topics, "patient,infect dis,infectious disease,bacterial meningitis,infection,treatment,streptococcus") AND LIMIT-TO(topics, "bacterial meningitis,treatment,streptococcus").

**EMBASE (Ovid)**

(("bacterial meningitis" or "bacterial meningitis") and "treatment").mp. [mp=title, abstract, heading word, drug trade name, original title, device manufacturer, drug manufacturer, device trade name, keyword, floating subheading word], limit 1 to (full text and human)

**CINAHL Plus**

bacterial meningitis OR bacterial meningitis AND treatment

Full Text

Age Groups: All Adult

**Cochrane Library**

bacterial meningitis or bacterial meningitis and "treatment"

Trials

**Global Health**

((bacterial meningitis) OR (bacterial meningitis) AND (treatment)) AND ( ((item-type:(( "Bulletin article" OR "Journal article" OR "Journal issue" OR "Standard" ) )) ))

**SciELO Citation Index**

TOPIC: (bacterial meningitis OR bacterial meningitis and treatment)

Refined by: SciELO Categories: ( INFECTIOUS DISEASES OR MEDICINE GENERAL INTERNAL OR PUBLIC ENVIRONMENTAL OCCUPATIONAL HEALTH OR TROPICAL MEDICINE ) AND DOCUMENT TYPES: ( RESEARCH ARTICLE OR UNDEFINED )

Indexes=SCIELO Timespan=All years

**Scopus**

( bacterial AND meningitis OR bacterial AND meningitis AND treatment ) AND ( LIMIT-TO ( EXACTSRCTITLE , "Clinical Infectious Diseases" ) OR LIMIT-TO ( EXACTSRCTITLE , "Antimicrobial Agents And Chemotherapy" ) OR LIMIT-TO ( EXACTSRCTITLE , "Journal Of Antimicrobial Chemotherapy" ) OR LIMIT-TO ( EXACTSRCTITLE , "Scandinavian Journal Of Infectious Diseases" ) ) AND ( LIMIT-TO ( DOCTYPE , "ar" ) ) AND ( LIMIT-TO ( SUBJAREA , "MEDI" ) OR LIMIT-TO ( SUBJAREA , "IMMU" ) OR LIMIT-TO ( SUBJAREA , "PHAR" ) OR LIMIT-TO ( SUBJAREA , "NEUR" ) OR LIMIT-TO ( SUBJAREA , "NURS" ) OR LIMIT-TO ( SUBJAREA , "HEAL" ) ) AND ( LIMIT-TO ( EXACTKEYWORD , "Human" ) OR LIMIT-TO ( EXACTKEYWORD , "Humans" ) OR LIMIT-TO ( EXACTKEYWORD , "Bacterial Meningitis" ) )

| Keywords: “bacterial meningitis” OR “bacterial meningitis AND treatment” | | |
| --- | --- | --- |
| Up to 8 February 2018 | | 9 February2018-9 March 2020 |
| Databases | HITS | HITS |
| CINAHL Plus | 49 | 2 |
| Cochrane Library | 512 | 40 |
| EMBASE | 540 | 1 |
| Global Health | 2722 | 243 |
| Ovid Medline | 353 | 7 |
| PubMed | 3768 | 16 |
| SciELO Citation Index | 82 | 6 |
| Science Direct | 1885 | 311 |
| Scopus | 394 | 62 |
| HITs | 10,305 | 688 |
| Duplicates | 2307 | 36 |
| Total HITs | 7998 | 652 |
| Total HITs | 8,650 | |

## eFigure 1.1 Flow diagram of search strategy and study selection.

Additional records identified through other sources
(n = 3)

8,614 Records excluded (did not meet inclusion criteria)

Records after title and abstract screening
(n = 39)

Records after duplicates removed
(n = 2,343)

Records identified through database searching (n = 10,993)

**Screening**

**Identification**

Records after full-text articles assessed for eligibility (n = 9)

Excluded 30 articles:

14 In pediatrics or patients mean age < 16 years or no mean age provided

1 Study on Pharmacokinetics/dynamics

6 In other infections/diseases

2 Overlapping data with included studies

1 Not RCT

1 No pharmacological intervention

2 No information on treatment interventions a/o clinical outcomes comparison between treatments

2 In immunocompromised population

1 Full-text could not be retrieved

1 autopsy report with overlapping data

1 letter to the editor

**Eligibility**

RCTs included in qualitative synthesis
(n = 9)

**Included**

6 RCTs included in quantitative synthesis (network meta-analysis) (n=6)

PRISMA, Preferred Reporting Items for Systematic Reviews and Meta-Analyses; RCT, randomized controlled trial

# eTable 1.1 Details of 30 studies excluded after full-text review

| **Reason for exclusion** | **Author** | **Year** |
| --- | --- | --- |
| **Studies in pediatrics or majority of participants are pediatrics** | Bryan, J, et. al. (1) | 1985 |
|  | Rucián, AF, et. al. (2) | 2011 |
|  | Girgis, N, et. al. (3) | 1972 |
|  | Girgis, N, et. al.(4) | 1988 |
|  | Girgis, N, et. al. (5) | 1989 |
|  | Ivler, D, et. al. (6) | 1963 |
|  | Nathan, NT, et. al. (7) | 2005 |
|  | Mathies, AW, et. al. (8) | 1965 |
|  | Overturf, GD, et. al. (9) | 1977 |
|  | Pecoul, B, et. al. (10) | 1991 |
|  | Pecoul, B, et. al. (11) | 1994 |
|  | Thomas, R, et. al. (12) | 1996 |
|  | Bademosi, O, et. al. (13) | 1976 |
|  | Wali, SS, et. al. (14) | 1979 |
| **Study on Pharmacokinetics/dynamics** | Buke, A, et. al. (15) | 2003 |
| **In other infections/diseases which are not in acute bacterial meningitis** | Chaudhary, M, et. al. (16) | 2008 |
|  | Fujii, R, et. al. (17) | 1979 |
|  | Hoepelman, I, et. al. (18) | 1988 |
|  | Marra, F, et. al. (19) | 1998 |
|  | Erdem, H, et. al. (20) | 2013 |
|  | Bennett, IL, et. al. (21) | 1963 |
| **Overlapping data with included studies** | Fritz, D, et. al. (22) | 2012 |
|  | Mai, NT, et. al. (23) | 2008 |
| **Not randomized controlled trial** | Girgis N, et. al. (3) | 1972 |
| **No pharmacological intervention** | MacFarlane, J. T., et. al. (24) | 1977 |
| **Study in HIV positive or immunocompromised patients** | Scarborough, M, et. al. (25)  *Note:* 90% of included patients were HIV positive. | 2008 |
|  | Richard, G. et. al. (26) | 2013 |
| **No information on treatment interventions a/o clinical outcomes comparison between treatments** | Bodilsen, J, et. al. (27) | 2014 |
|  | Wang, S-P, et. al. (28) | 2007 |
| **Full-text could not be retrieved** | Gupta A & Singh N. (29) | 1996 |

# Appendix 2

## eTable 2.1 Detail of antibiotic regimens

| **Abbreviation** | **Description** |
| --- | --- |
| CHLO | Chloramphenicol |
| CEP | Cephalosporins include ceftriaxone, cefepime, ceftazidime, cefotaxime |
| CS | Corticosteroids include dexamethasone or other adjunct corticosteroid use in combination with antibiotics |
| MPN | Meropenem |
| PEN | Penicillin antibiotics include natural penicillins e.g. penicillin G and Aminopenicillins e.g. amoxicillin, ampicillin |
| VAN | Vancomycin in conventional dose (15 mg/kg q 12 h) |
| HVAN | High dose vancomycin (15 mg/kg q 8 h) |
| CEP+VAN | Cephalosporin in combination with vancomycin |
| CS+CEP | Corticosteroid in combination with one of cephalosporins |
| CS+PEN | Corticosteroid in combination with one of penicillin antibiotics |
| PEN+CHLO | One penicillin antibiotic in combination with chloramphenicol |
| CS+CEP+VAN | Corticosteroid in combination with one of one of cephalosporins and vancomycin |
| CS+PEN+CHLO | Corticosteroid in combination with one of Penicillin antibiotics and Chloramphenicol |

# Appendix 3

**Description of included studies**

## eTable 3.1 Key characteristics of included studies

| **Authors (year)** | **Country** | **Study design** | **Main pathogen (N)** | **Intervention** | **Comparator** | **Study size** | **Age per group (mean±SD)** | **Male (%)** | **Primary outcomes** | **Adverse events** |
| --- | --- | --- | --- | --- | --- | --- | --- | --- | --- | --- |
| CS+ABT vs. Double | | | | | | | | | |  |
| Bhaumik, S. (1998)(30) | India | A single center, randomized clinical trial | *N. meningitis* (n=6) *S. pneumoniae* (n=9) *Note:* No of isolates =15 | Dexamethasone (4 mg q 6 h 4 days then 4 mg tid on 5th day, bid on day 6th-7th) i.v. plus antibiotics ≥ 10 days (CS+PEN+CHLO)(n=14) *Note:* 13 patients were initially treated with C-pen (20 lac units IV q 4)+Chloramphenical (1 g IV q 6 h) Dexamethasone was started after 1^st^ dose of antibiotics | Antibiotics ≥ 10 days (PEN+CHLO) (n=16) *Note*: 14 patients were initially treated with C-pen (20 lac units IV q 4)+Chloramphenical (1 g IV q 6 h) | 30 | 28±17 vs. 34±19 | 13 (92.8) vs. 13 (81.3) | Death 1 (7.1) vs. 3 (18.8), p=0.60 Neurological sequelae: 3 (21.4) vs. 2 (12.5), p = 0.64 Audiological sequelae*: 4 (28.5) vs. 3 (18.75), p=1.00 *Note*: * Assessed by brainstem auditory evoked responses a/o pure tone auditory | NR |
| CS+ABT vs. Mono | | | | | | | | | |  |
| de Gans, J* (2002)(31) | Europe (The Netherlands, Belgium, Germany, Denmark, Austria) | A prospective, randomized, double-blind, multicenter trial | *S. pneumoniae* (n=108, 36%) *Neisseria meningitis* (n=97, 33% Others (n=29, 9.7%) Negative CSF culture (n=65, 21.3%)  *Note:* CSF culture was performed in 299 patients (155 vs. 144) | Dexamethasone sodium phosphate 10 mg q 6 h iv, 4 dys 15-20 mins before or with antibiotics (CS+PEN) (n=157)  *Note*: Most patients initially received amoxicillin 2 g iv q 4 h 7-10 dys 77% patients received amoxicillin and penicillin, 8% 3^rd^ generation cephalosporin, 8% amoxicillin or penicillin + cephalosporin | Placebo+antibiotic (PEN) (n=144)  *Note:* Most patients initially received amoxicillin 2 g iv q 4 h 7-10 dys | 301 | 44±18 vs  46±20 | 89 (57) vs 80 (56) | RR of death, 95%CI: 0.48 (0.24-0.96), p = 0.04  RR of an unfavorable outcome in intervention to comparator, 95%CI: 0.59 (0.37-0.394), p = 0.03  Focal neurologic abnormalities, 18/143 (13) vs. 24/119 (20); 95%CI: 0.62 (0.36-1.09), p=0.13  Hearing loss 13/143 (9) vs. 14/119 (12); 95%CI: 0.77 (0.38-1.58), p=0.54 | Gastrointestinal bleeding 2 (1) vs. 5 (3), p=0.27  Hyperglycemia 50 (32) vs. 37 (26), p =0.24  Herpes zoster 6 (4) vs. 4 (3), p =0.75  Fungal infection 8 (5) vs. 4 (3), p=0.38 |
| Gijwani, D (2002)(32) | India | A prospective placebo controlled, randomized double-blind study | *Pneumonococci* (n=8, 40%) *Staphylococci* (n=6, 30%) *Streptococci* (n=4, 20%) *H influenzae Streptococci* (n=4, 20%) *Meningococci* (n=2, 10%) E. Coli *Meningococci Streptococci* (n=2, 10%) | Dexamethasone (0.6 mg/kg/dy q 6 h first 4 days)+ceftriaxone 100 mg/kg/dy 14 days (CS+CEP) (n=20) *Note:* Dexamethasone was given at least 15 mins before ceftriaxone | Placebo+ ceftriaxone 100 mg/kg/dy 14 days (CEP) (n=20) | 40 | 28.25 ± 16.75 vs 32.25 ± 1.64 | 11 (55) vs 15 (75) | Mortality 2/18 (11.12) vs. 4/16 (25) Neurological sequelae at 90th day: 2/18 (11.12) vs. 4/16 (25) Hearing loss at 90th day: 7/18 (38.89) vs. 9/16 (56.25) | Gastrointestinal bleeding 3/20 (15%) vs. 1/20 (5%), p=<0.05  Secondary fever 3/20 (15%) vs. 1/20 (5%), p<0.05  Psychiatric manifestations 2/20 (10%) vs. 0, p <0.05 |
| Nguyen, TH (2007)(33) | Vietnam | A randomized, double-blind, placebo-controlled trial | *S. suis* (n=116, 52.3%) *S. pneumoniae* (n=55, 24.8%) *Streptococcus species* (n=18, 8.1%) *S. Aureus* (n=9, 4.1%) *N. meningitidis* (n=19, 8.6%) *H. influenzae* (n=7, 3.2%) *Klebsiella species* (n=10, 4.5%) *E. coli* (n=9, 4.1%) Other -ve bacteria (n=4, 1.8%) | Dexamethasone 0.4 mg/kg q 12 h, 4 dys 15 mins before antibiotics + ceftriaxone 2 g iv, q 12 h, 10-14 dys (CS+CEP) (n=217, 143*)  *Note:* Antibiotic treatment could be altered based on physician's discretion *Definite bacterial meningitis: If bacteria were detected in CSF or blood culture at the time of discharge or death | Placebo+ ceftriaxone 2 g iv, q 12 h, 10-14 dys (CEP) (n=218, 157*)  *Note:* Antibiotic treatment could be altered based on physician's discretion *Definite bacterial meningitis: If bacteria were detected in CSF or blood culture at the time of discharge or death | 435 300* | 42 (15-89) vs. 41 (15-91) *Note:* Median | 165/217 (76) vs. 152/218 (69.7) | Death 1 month after randomization:18/217 (8.29) vs. 26/218 (11.93)  All patients: - RR of death at 1 months = 0.79 (0.45-1.39),ns - RR of death or disability at 6 months = 0.74 (0.47-1.17),ns  Definite BM: - RR of death at 1 month = 0.43 (0.20-0.94), p=0.03 - RR of death or disability at 6 months = 0.56 (0.32-0.98), p=0.03 | Minor gastrointestinal bleeding 10/217 (4.6%) vs. 5/218 (2.3%), p=0.20  *Note:* major gastrointestinal bleeding in 1 patient each at both group  Herpes labialis 33 (15.2%) vs. 30 (13.8%), p=0.69 |
| Thomas, R (1999)(34) | France and Switzerland | Multicenter, double-blind, randomized trial | *S. pneumoniae* (n=31, 52%) *N. menigitidis* (n=18, 30%) Unknown (n=8, 13.3%) Others (n=3, 5%) | Aminopenicillin+ dexamethasone 10 mg qid 3 days (within 3 hours after aminopenicillin therapy initiation) (CS+PEN) (n=31)  *Note:* the first dose of dexamethasone was given within 3 hours after initiation of antibiotics | Aminopenicillin+placebo qid 3 days (within 3 hours after aminopenicillin therapy initiation) (PEN) (n=29) | 60 | 40±19 vs 50±19 | 55 vs 59 | The rate of patients cured without neurological sequelae at day 30: 23 (74) vs. 15 (52), p. 0.0711  Mild neurological sequelae at day 30: 2 (6.45%) vs. 4 (13.79%)  Severe neurological sequelae: 3 (9.68%) vs. 5 (17.24%) | Pain at injection 1 vs. 0  Transient hyperglycemia 1 vs. 0  Gastric ulcer with overt hemorrhage 0 vs. 2  Herpes zoster 0 vs. 1 |
| Mono vs. Double | | | | | | | | | |  |
| Zavala, I (1988)(35) | Mexico | An open, randomized comparative study | *S. pnuemoniae* (n=13) *S. epidermidis* (n=3) *H. influenza* (n=2) *E. coli* (n=6) *S. typhi* (n=1) | Ceftriaxone i. v. 4 g OD (CEP) (n=13) *Note:* Dose decreased to 2 g when CSF became sterile | Ampicillin+ Chloramphenicol i.v. (PEN+CHLO) (n=13) *Note*: Ampicillin dose 200-400 mg/kg/dy, chloramphenicol dose 2-3 g/dy in 4 divided doses | 26 | 28.6 (14-51) vs. 25.3 (16-52) | 61.5 vs. 69.2 | The overall clinical and bacteriological cure rate (CSF sterile after 10 days): 100% (13/13) vs. 92% (12/13)* *Note:* *1 patient withdrew due to treatment failure | Skin rashes 0 vs. 3  Diarrhea 0 vs. 3 |
| Others | | | | | | | | | |  |
| Elyasi, S (2015)(36) | Iran | A randomized, open-labeled study | *S. pneumoniae* (n=25, 56.82%) *MRSA* (n=2, 4.54%) *S. epidermidis* (n=1, 2.27%) *E. faecalis* (n=1, 2.27%) *Note:* From 29/44 (65.9%) who had positive CSF culture | Vancomycin 15 mg/kg q 8 h (high dose)+Ceftriaxone 2g q 12h (n=22)  *Note*: All patients received 1st dose of antibiotics within 1 h of hospital admission. Most common regimen: Vancomycin plus ceftriaxone 2 g q 12 h (20 vs. 20) Target trough level = 15-20 mg/ml for ABM | Vancomycin 15 mg/kg q 12 h (conventional-dose)+ Ceftriaxone 2g q 12 h (n=22) *Note:* All patients received 1st dose of antibiotics within 1 h of hospital admission Target trough level = 15-20 mg/ml for ABM | 44 | 50.56±18.22 vs 46.13±17.11, p=0.22 | 68 (13) vs 59 (15) | GCS at 10th dy: 11 vs. 13, p = 0.02  CrCL at 10th day 102.14±44.24 vs 98.99±13.87 ml/min, p = 0.65 Time to normal WBC 3.22±3.11 vs 6.00±2.45, p=0.03 Time to afebrile 3.35±1.23 vs 6.11±2.00, p=0.02 Duration of hospitalization day: 12.85±5.47 vs 10.10±2.45, p = 0.04 | NR |
| Narciso, P (1983)(37) | Italy | A single-center, randomized controlled trial | *N. meningitidis* (n=3) E. coli (n=1) *D. pneumoniae* (n=3) *Note:* Negative culture in 3 patients | Ceftriaxone i.v. q 12 h 100-80 mg/kg/day in 4 cases and 45 mg/kg in 1 case (CEP) (n=5) | Ampicillin (110 mg/kg) divided in 3 slow iv q 8 hr (PEN) (n=5) | 10 | 48 vs. 44 | NR | All patient recovered completely | NR |
| Schmutzhard, E (1995)(38) | Hungary, the Czech Republic, Portugal, France, Spain, Austria (from 15 centres) | Two prospective randomized controlled studies | *H. influenzae* 7.1% (n=1) *N. menigitidis* 39.3% (n=18) *S. pneumoniae* 78.6% (n=31) Others 3.57% (n=2) | Meropenem 40mg/kg q 8 h, up to a maximum of 6g/dy, 7-14 dys¶ (MPN) (n=28)  *Note*: Dexamethasone was administered to 39 patients (meropenem 19, cephalosporins 20) | Cephalosporins, 7-14 dys¶ (n=28): Cefotaxime (n=17) Ceftriaxone (CEP) (n=11)  *Note*: Dexamethasone was administered to 39 patients (meropenem 19, cephalosporins 20) | 56 | 46 (17-76) vs. 31 (13-71) *Note*: Median | 11(39.3) vs. 17 (60.7) | Clinical cured: Meropenem vs. Cephalosporins: 23/23 (100%) vs. 17/22 (77%)  Neurological sequelae: Meropenem 3 (10.7%) vs. Cephalosporins 4 (14.3%); 4 receiving cefotaxime  Hearing impairment: 11 (39.3%) vs. 9 (32%)  Death: Meropenem 3 vs. 1 Cephalosporins | Oral candidiasis 3 vs. 0  Rash and neuropathy 1 vs. 0  Inflammation at injection site 1 vs. 2  Transient elevated ALT 2 vs. 3  Transient elevated AST 2 vs. 0  Prolonged prothrombin time 1 vs. 0  Thrombocytosis 0 vs. 1  Eosinophilia 0 vs. 1 |

**Abbreviations:** Mono: Mono antibiotic therapy; Double: Dual antibiotic therapy; CS: Corticosteroids; ABT: Antibiotic(s); BM: Bacterial meningitis; C-pen: crystalline penicillin; CSF: Cerebrospinal fluid; FU: follow-up; GCS: GCS: Glasgow Coma Scale score; i.m.: intramuscular; i.v.: intravenous; OD: once daily; SAPS 1: the Simplified Acute Physiologic Score; NR: Not reported; ALT: Alanine transaminase; AST: Aspartate transaminase

**Notes:** ^a^ Cure: complete resolution of signs and symptoms of meningitis without other antibiotics addition; ^b^ Unfavourable outcome: GCS 1-4; ¶ Patients were randomized to either intravenous treatment with meropenem (40mg/kg q 8 h, up to a maximum of 6g/dy) or ceftriaxone (an initial dose of 100 mg/kg followed by a single daily dose of 80 mg/kg up to a maximum of 4g/dy), cefotaxime 75-100 mg/kg q 8 h (225-300 ng/kg/dy up to a maximum of 12g/dy); *>75% same type of antibiotic prescribed

## eTable 3.2 Description of study participants

| **First Author** | **Year** | **Inclusion** |
| --- | --- | --- |
| Bhaumik, S. | 1998 | Patients with bacterial meningitis aged between 12-75 yrs admitted to the Neurology department who fulfilled any following criteria:   1. CSF WBC count >100 per mm^3^, with at least 60% polymorphs, increased protein and decreased sugar less than half of blood sugar level in CSF 2. Clinical picture suggestive and identification of organism of CSF by culture or Gram staining |
| de Gans, J | 2002 | Patients ≥17 yrs suspected meningitis in combination with cloudy cerebrospinal fluid/bacteria in CSF on Gram's staining, CSF leukocyte count ≥1000 per mm^3^ |
| Elyasi, S | 2015 | Diagnosis of acute bacterial meningitis based on clinical signs and symptoms plus either positive CSF or at least 2 of CSF protein > 100 mg/dl, glucose concentration < 50 mg/dl or <50% of serum glucose or elevated WBC (> 500 cells/µl) |
| Gijwani, D | 2002 | Established cases of pyogenic meningitis of ≥ 10 years with at least one of the following criteria:   1. Turbid or purulent CSF with identification of bacteria on Gram staining or CSF culture 2. CSF leukocytosis (>10 to <10,000 per mm^3^) with neutrophils predominance 3. Elevated CSF protein above 50 mg/dl and glucose < 40 mg% |
| Narciso, P | 1883 | Adult patients suffering from purulent meningitis |
| Nguyen, TH | 2007 | Patients aged > 14 yrs who had suspected bacterial meningitis Clinical evidence of meningitis and at least 1 of followings: 1. Bacteria detected in CSF or blood culture 2. Clinical history ≤ 7 days of illness with a cloudy CSF, WBC count > 60% neutrophils and a ratio of CSF to blood glucose < 50% |
| Schmutzhard, E | 1995 | Patients hospitalized with clinical signs and symptoms of BM, requiring intravenous antibiotics and had CSF pathogen likely to be susceptible to both meropenem and cephalosporin comparators |
| Thomas, R | 1999 | Adults aged 18-79 yrs, admitted to one of 21 participating ERs/ICUs with clinical signs of bacterial meningitis |
| Zavala, I | 1988 | Adults showing clinical evidence of acute bacterial meningitis, confirmed by CSF |

**Abbreviations:** BM: Bacterial Meningitis; CSF: Cerebrospinal fluid; ER: Emergency Room; ICU: Intensive Care Unit; WBC: White blood cell count; yrs: years

**Note:** Inclusion criteria are excerpts or summaries from included articles.

#

# Appendix 4

**Risk of bias assessment**

## eFigure 4.1 Summarized risk of bias of included randomized controlled trials (RoB2.0)


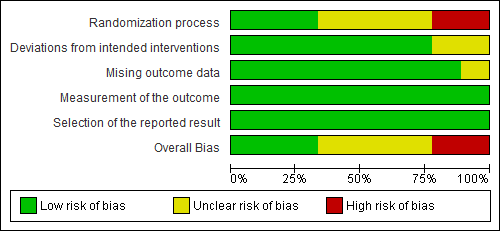


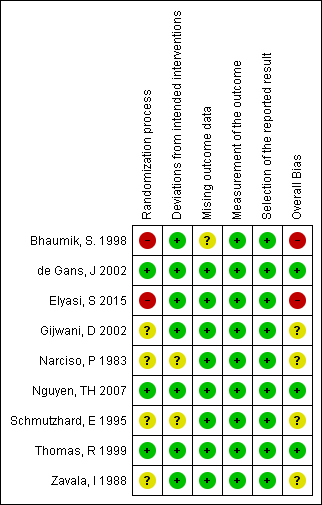


# Appendix 5

**Network plot of all treatment comparisons**

## eFigure 5.1 all-cause mortality


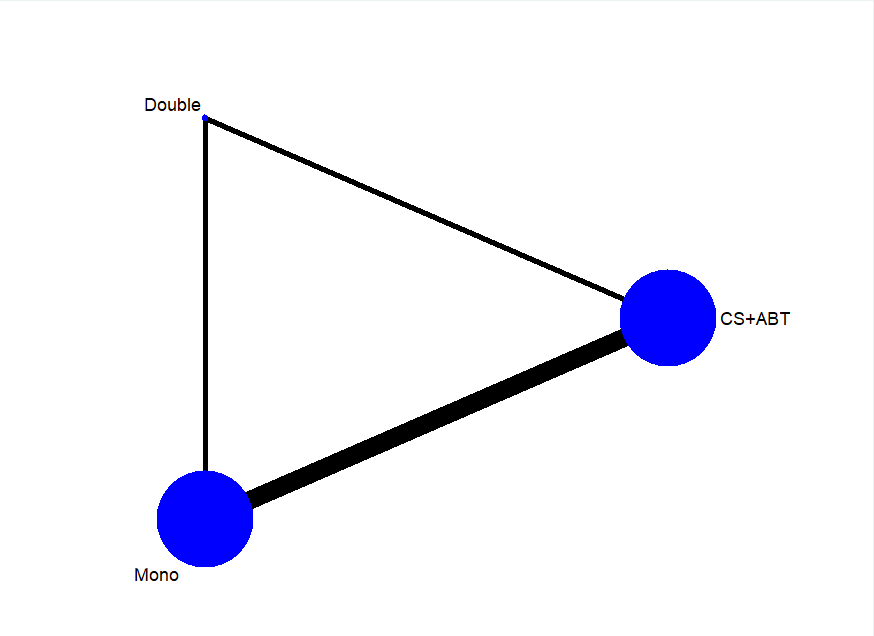


## eFigure 5.2 Neurological sequelae

##
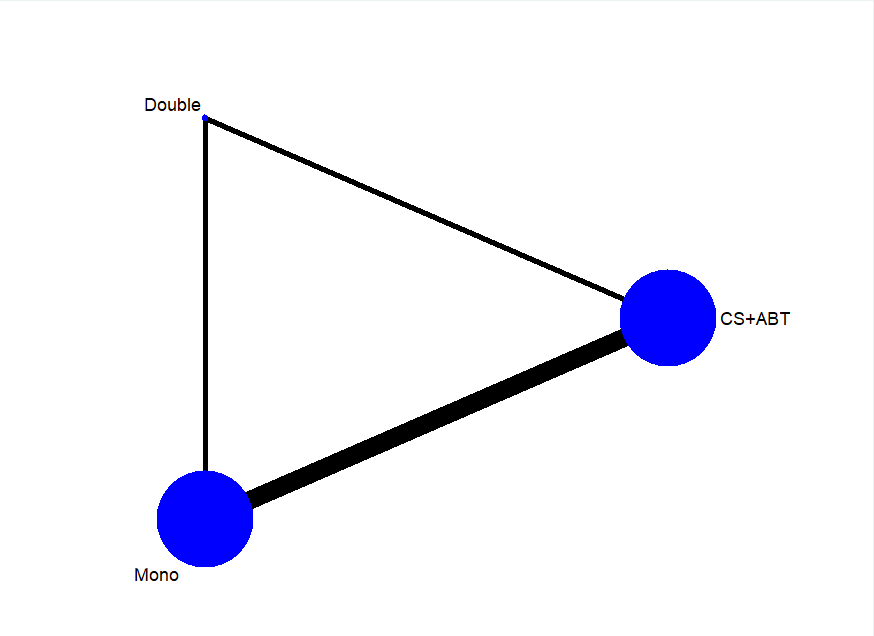


## eFigure 5.3 Any hearing loss


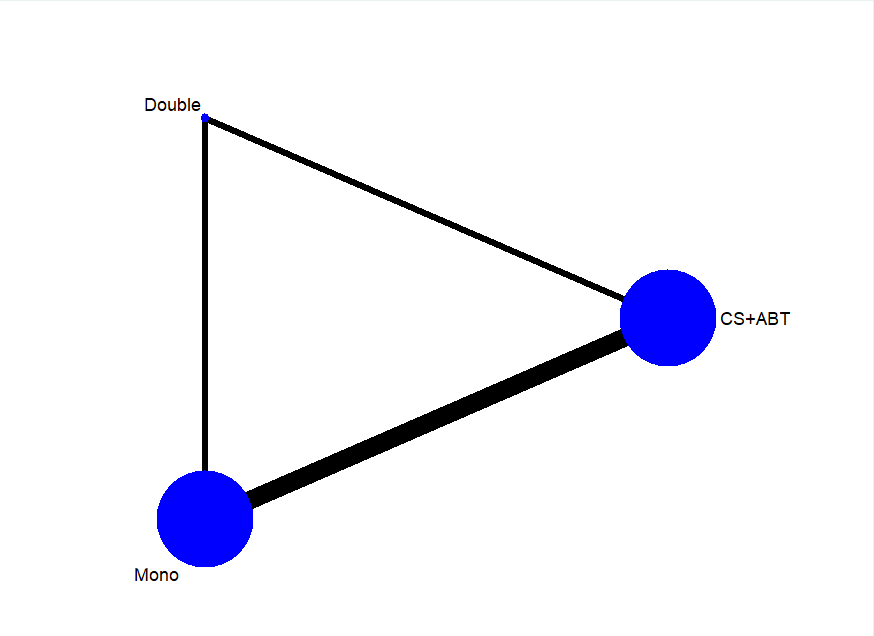


The node size corresponds to the number of studies examining the interventions. Each direct treatment comparisons are linked with a line. The line thickness represents the number of studies assessing the comparison.

Abbreviations: Mono, Mono antibiotic therapy; Double, Dual antibiotic therapy; CS, Corticosteroids; ABT, Antibiotic(s)

# Appendix 6

**Assessment of inconsistency in intervention triangular loops for each outcome network**

## eFigure 6.1 Assessment of global inconsistency in networks using the ‘design-by-treatment’ interaction model.

1. **All-cause mortality**

**
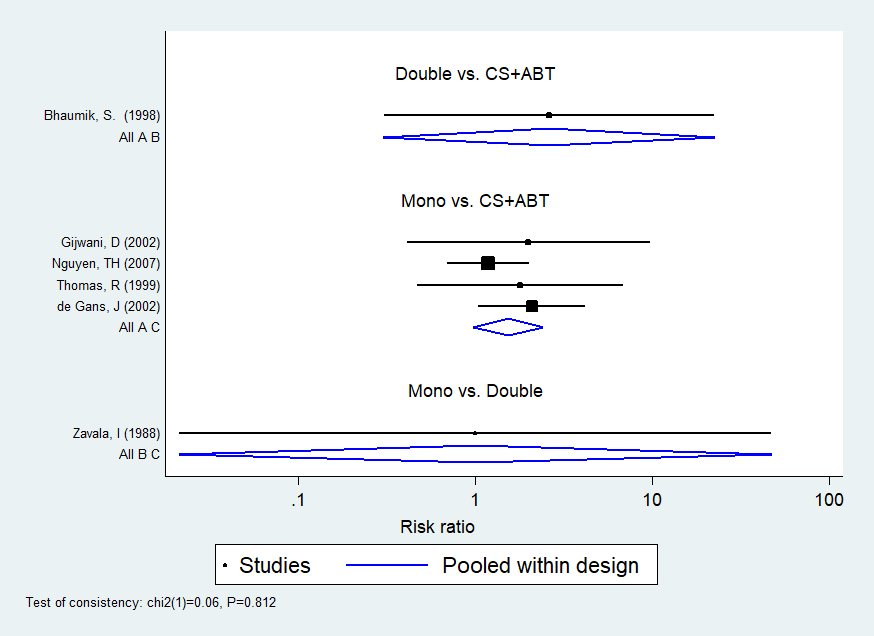
**

1. **Neurological sequelae**

**
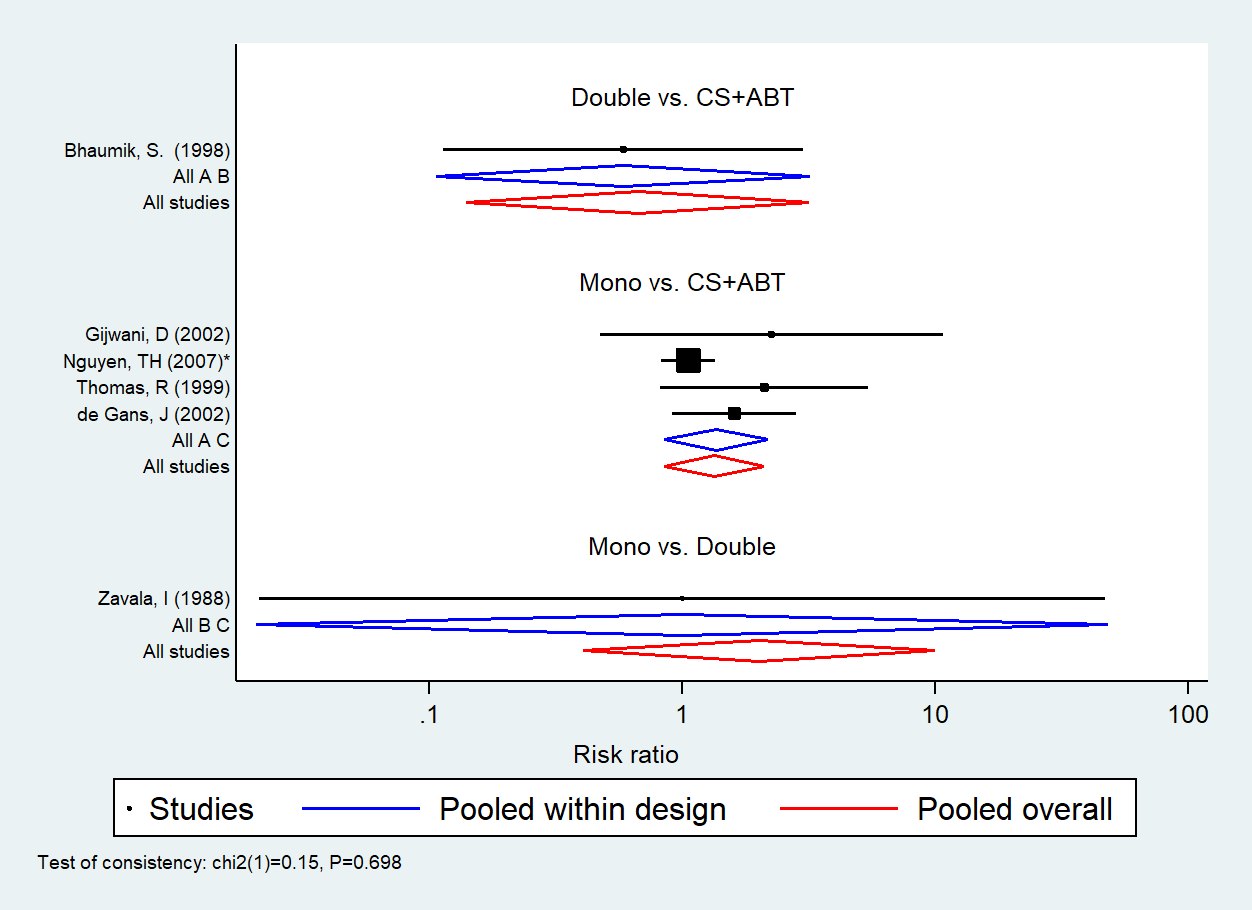
**

1. **Any hearing loss**

**
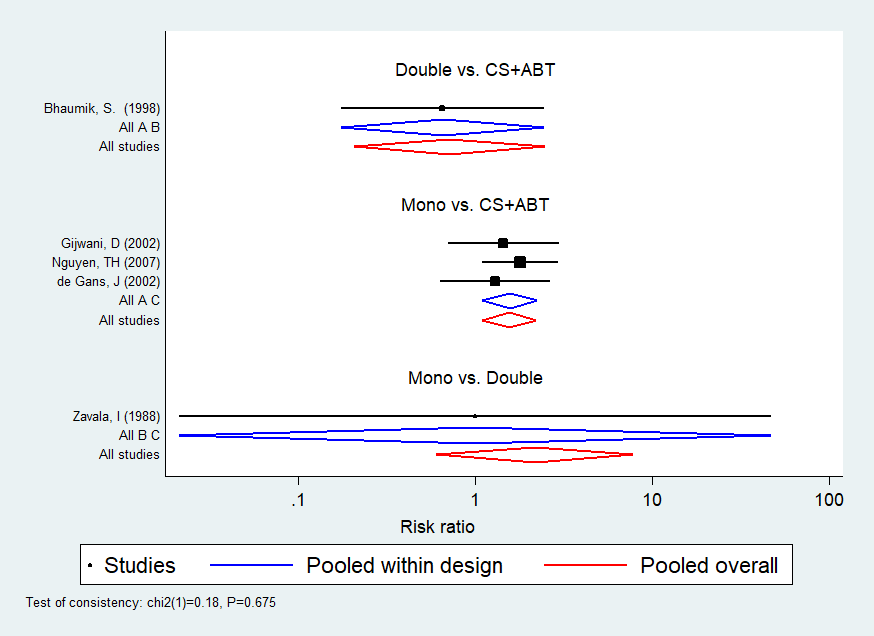
**

## eTable 6.1 Assessment of global inconsistency in networks using the ‘design-by-treatment’ interaction model.

| **Network outcome** | **Chi-square** | **P value for test of global inconsistency** |
| --- | --- | --- |
| All-cause mortality | 0.06 | 0.8117 |
| Neurological sequelae | 0.15 | 0.6980 |
| Hearing loss | 0.18 | 0.6751 |

## eTable 6.2 Inconsistency test between direct and indirect comparisons in mixed treatment comparison for all-cause mortality

| Side | Direct | | Indirect | | Difference | | p>z |
| --- | --- | --- | --- | --- | --- | --- | --- |
|  | **Coefficient** | **SE** | **Coefficient** | **SE** | **Coefficient** | **SE** |  |
| A C | .4250253 | .2299505 | .9648633 | 2.255029 | -.539838 | 2.266723 | 0.812 |
| A B | .9650845 | 1.10228 | .4250468 | 1.981184 | .5400377 | 2.267224 | 0.812 |
| B C | -2.83e-12 | 1.967937 | -.5400248 | 1.12595 | .5400248 | 2.267276 | 0.812 |

## eTable 6.3 Inconsistency test between direct and indirect comparisons in mixed treatment comparison for neurological sequelae

| Side | Direct | | Indirect | | Difference | | p>z |
| --- | --- | --- | --- | --- | --- | --- | --- |
|  | **Coefficient** | **SE** | **Coefficient** | **SE** | **Coefficient** | **SE** |  |
| A C | .3051328 | .2416212 | -.5387446 | 2.160749 | .8438774 | 2.174215 | 0.698 |
| A B | -.5389989 | .8703256 | .3051861 | 1.993199 | -.844185 | 2.174912 | 0.698 |
| B C | -8.93e-13 | 1.978688 | .8440915 | .9032273 | -.8440915 | 2.175092 | 0.698 |

## eTable 6.4 Inconsistency test between direct and indirect comparisons in mixed treatment comparison for any hearing loss

| Side | Direct | | Indirect | | Difference | | p>z |
| --- | --- | --- | --- | --- | --- | --- | --- |
|  | **Coefficient** | **SE** | **Coefficient** | **SE** | **Coefficient** | **SE** |  |
| A C | .4519256 | .1806523 | -.4209391 | 2.073974 | .8728646 | 2.081827 | 0.675 |
| A B | -.4212165 | .6703761 | .4520523 | 1.97197 | -.8732688 | 2.08279 | 0.675 |
| B C | -3.92e-12 | 1.963961 | .873097 | .6942812 | -.873097 | 2.083067 | 0.675 |

SE, standard error; A, CS+ABT; B, Double; C, Mono

# Appendix 7

**Results of meta-analyses of direct comparisons**

## eFigure 7.1 Results of pairwise meta-analyses for all-cause mortality.


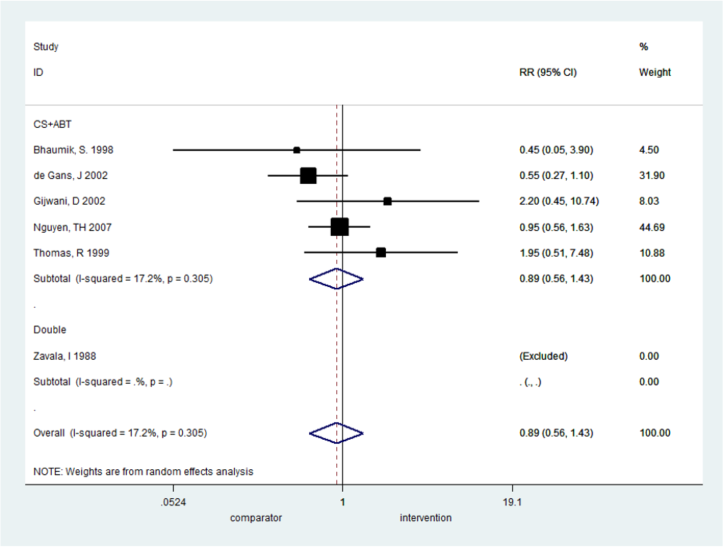


## eTable 7.1 Results of pairwise meta-analyses for all-cause mortality.

| **Comparisons** |  | **No. of studies** | **Pairwise meta-analysis rate ratio (95% CI)** | **Heterogeneity I^2^ (variation in RR attributable to heterogeneity)** |
| --- | --- | --- | --- | --- |
| **All-cause mortality** |  |  |  |  |
| CS+ABT vs. | Mono | 5 | 0.89 (0.56 to 1.43) | 17.2% |
| Double vs. | Mono | 1 | NA | NA |

Abbreviations: Mono, Monotherapy; Double, Dual therapy; CS, Corticosteroids; ABT, Antibiotics

## eFigure 7.2 Results of pairwise meta-analyses for neurological sequelae.


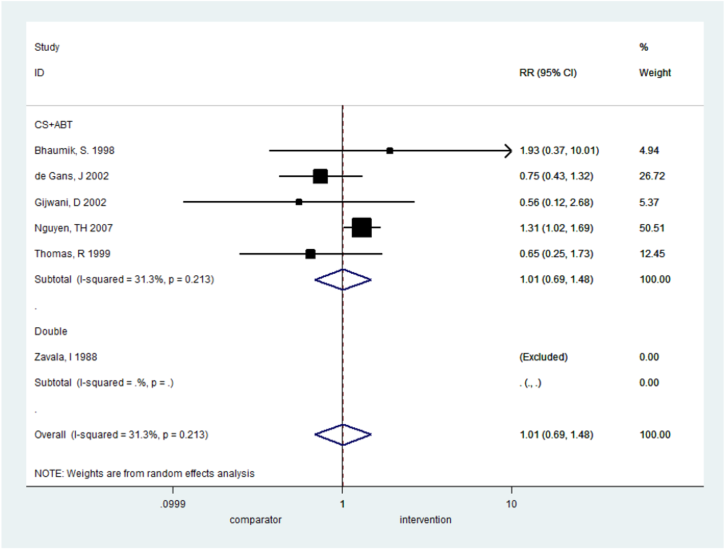


## eTable 7.2 Results of pairwise meta-analyses for neurological sequelae.

| **Comparisons** |  | **No. of studies** | **Pairwise meta-analysis rate ratio (95% CI)** | **Heterogeneity I^2^ (variation in RR attributable to heterogeneity)** |
| --- | --- | --- | --- | --- |
| **Neurological sequelae** |  |  |  |  |
| CS+ABT vs. | Mono | 5 | 1.01 (0.69 to 1.48) | 31.3% |
| Double vs. | Mono | 1 | NA | NA |

Abbreviations: Mono, Mono antibiotic therapy; Double, Dual antibiotic therapy; CS, Corticosteroids; ABT, Antibiotic(s)

## eFigure 7.3 Results of pairwise meta-analyses for any hearing loss.


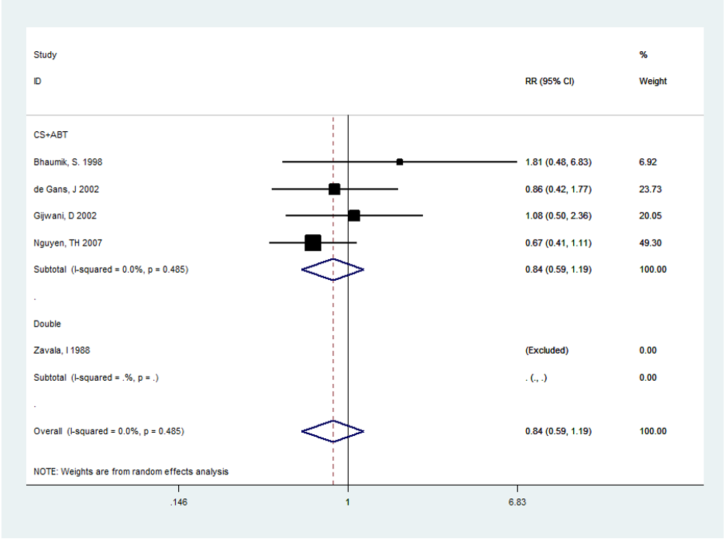


## eTable 7.3 Results of pairwise meta-analyses for any hearing loss.

| **Comparisons** |  | **No. of studies** | **Pairwise meta-analysis rate ratio (95% CI)** | **Heterogeneity I^2^ (variation in RR attributable to heterogeneity)** |
| --- | --- | --- | --- | --- |
| **Any hearing loss** |  |  |  |  |
| CS+ABT vs. | Mono | 4 | 0.84 (0.59 to 1.19) | 0% |
| Double vs. | Mono | 1 | NA | NA |

Abbreviations: Mono, Monotherapy; Double, Dual therapy; CS, Corticosteroids; ABT, Antibiotics

**eFigure 7.4 Results of network meta-analysis of treatment options**

1. **All-cause mortality**


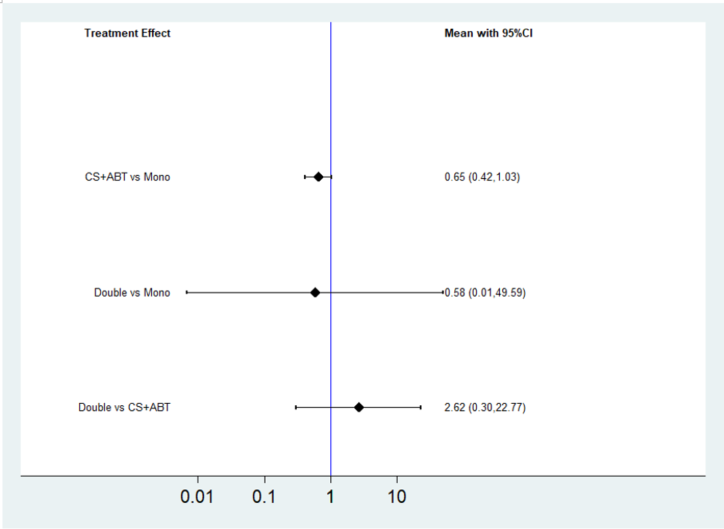


1. **Neurological sequelae**


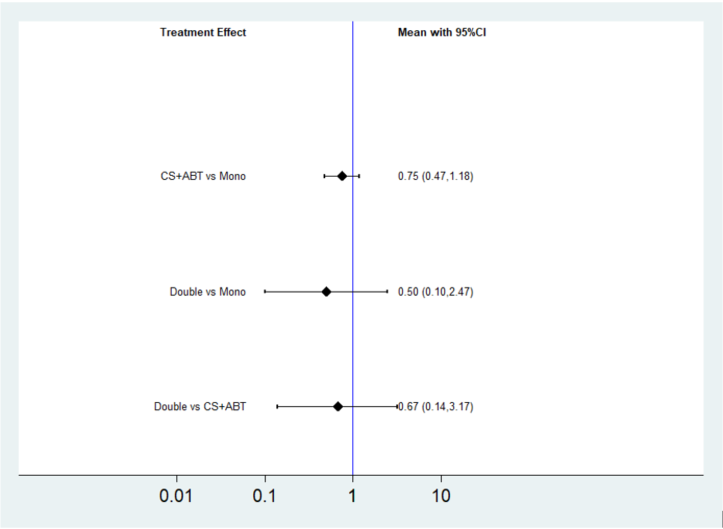


1. **Any hearing loss**


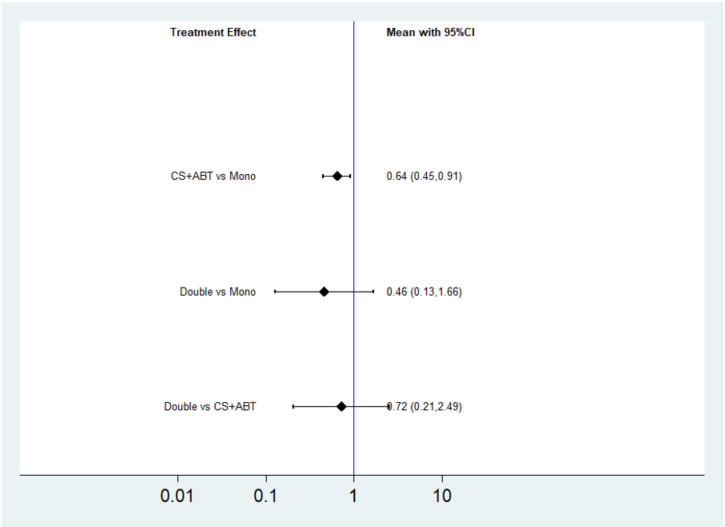


**eFigure 7.5 Contribution plots of treatment options**

1. **All-cause mortality**


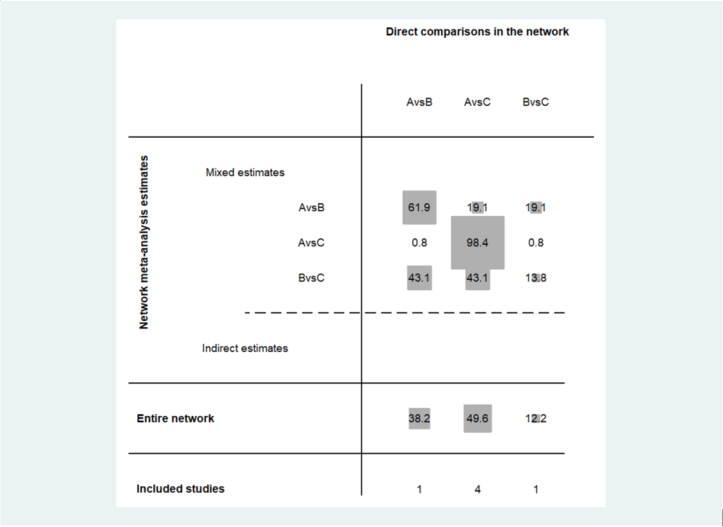


1. **Neurological sequelae**


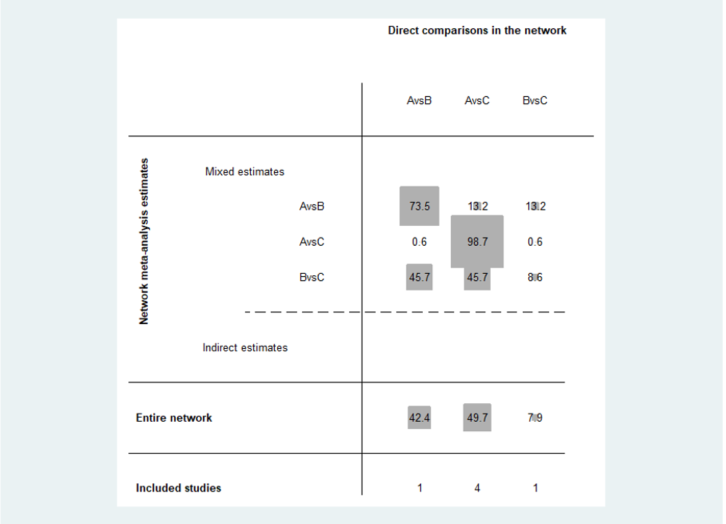


1. **Any hearing loss**


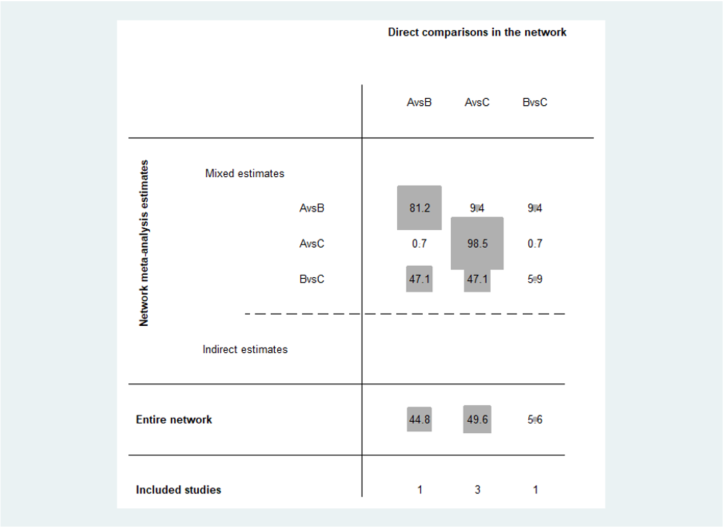


A, CS+ABT; B, Double; C, Mono

Abbreviations: Mono, Mono antibiotic therapy; Double, Dual antibiotic therapy; CS, Corticosteroids; ABT, Antibiotic(s)

# Appendix 8

**Results of network meta-analyses**

## eFigure 8.1 Network estimated rate ratios (95% confidence intervals) for all-cause mortality.

| CS+ABT |  |  |
| --- | --- | --- |
| 0.43 (0.07,2.86) | Double |  |
| 0.65 (0.42,1.02) | 1.50 (0.22,10.21) | Mono |

#

## eFigure 8.2 Network estimated rate ratios (95% confidence intervals) for neurological sequelae.

| CS+ABT |  |  |
| --- | --- | --- |
| 1.50 (0.32,7.13) | Double |  |
| 0.75 (0.47,1.18) | 0.50 (0.10,2.47) | Mono |

## eFigure 8.3 Network estimated rate ratios (95% confidence intervals) for any hearing loss.

| CS+ABT |  |  |
| --- | --- | --- |
| 1.39 (0.40,4.83) | Double |  |
| **0.64 (0.45,0.91)** | 0.46 (0.13,1.66) | Mono |

Abbreviations: Mono, Mono antibiotic therapy; Double, Dual antibiotic therapy; CS, Corticosteroids; ABT, Antibiotic(s)

# Appendix 9

**Treatment ranking and surface under the cumulative ranking (SUCRA) curves for each outcome**

## eFigure 9.1 SUCRA ranking curves for all-cause mortality.


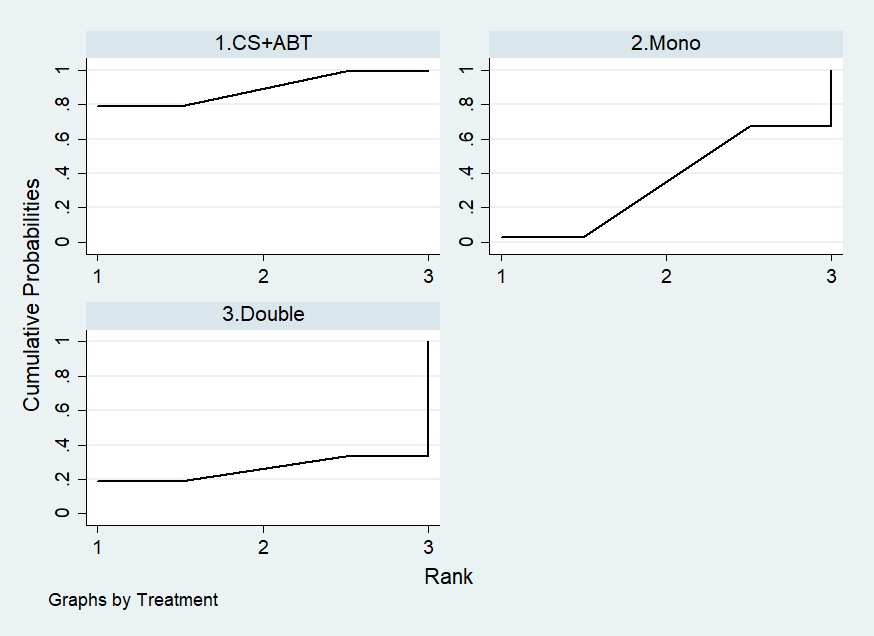


**26.1%**

**89.0% 35.0%**

Abbreviations: Mono, Mono antibiotic therapy; Double, Dual antibiotic therapy; CS, Corticosteroids; ABT, Antibiotic(s)

## eFigure 9.2 SUCRA ranking curves for neurological sequelae.


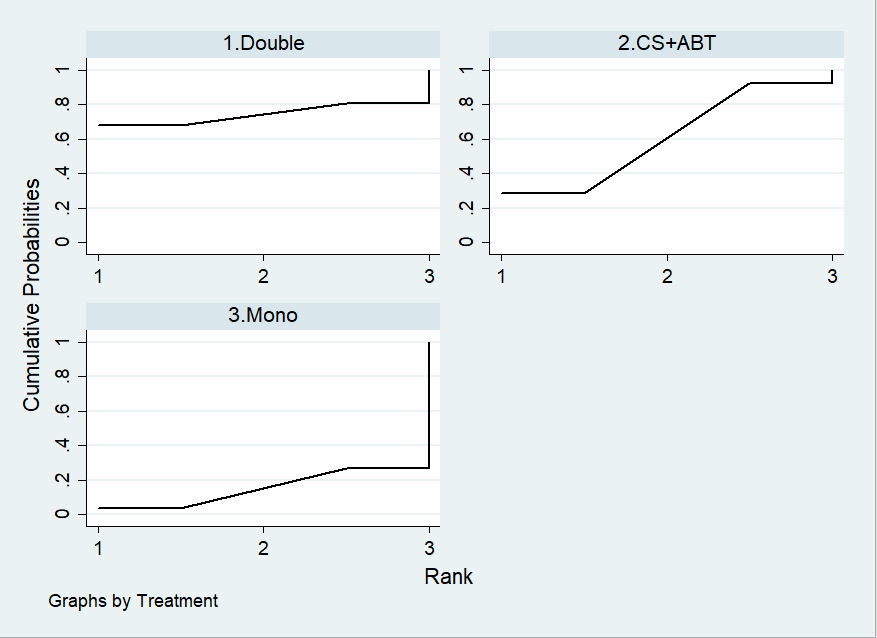


**74.5% 60.4%**

**15.0%**

Abbreviations: Mono, Mono antibiotic therapy; Double, Dual antibiotic therapy; CS, Corticosteroids; ABT, Antibiotic(s)

## eFigure 9.3 SUCRA ranking curves for any hearing loss

##
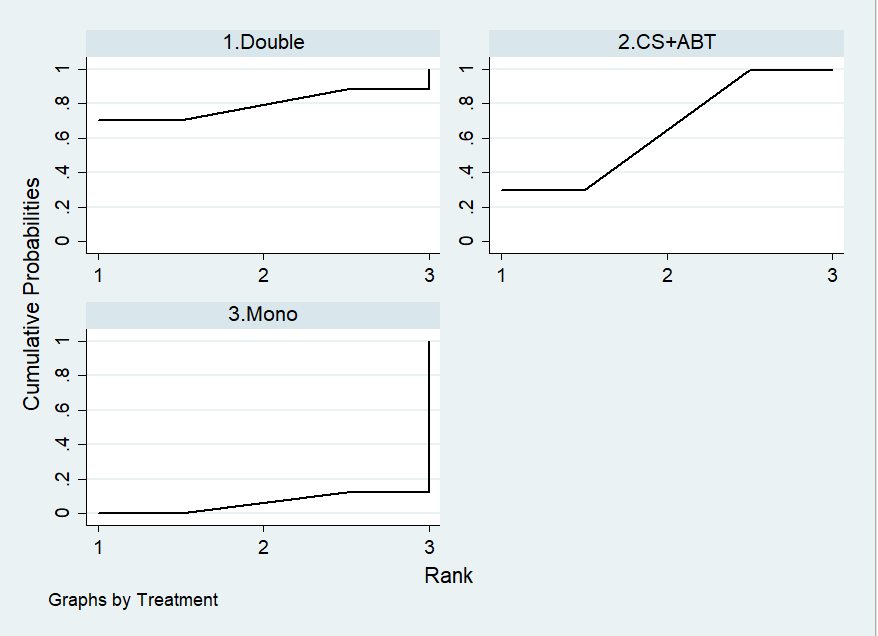


**79.3% 64.6%**

**6.1%**

Abbreviations: Mono, Mono antibiotic therapy; Double, Dual antibiotic therapy; CS, Corticosteroids; ABT, Antibiotic(s)

# Appendix 10

# Evaluation of the quality of evidence using GRADE framework for primary outcomes

**Table 10.1 Direct estimates and GRADE quality assessment of direct evidence for each pairwise comparison for primary outcomes**

| **Certainty assessment** | | | | | | **Effect** | | **Certainty** |
| --- | --- | --- | --- | --- | --- | --- | --- | --- |
| **Treatment** | **№ of study (n)** | **Risk of bias** | **Inconsistency** | **Indirectness** | **Imprecision** | **Relative (95%CI)** | **Absolute (95%CI)** |  |
| **All-cause mortality** | | | | | | | | |
| CS+ABT vs. Double | 1 (30) | serious ^a^ | not serious ^b^ | not serious | serious ^c^ | **RR 0.45** (0.05 to 3.9) | **103 fewer per 1,000** (from 178 fewer to 544 more) | ⨁⨁◯◯ LOW |
| CS+ABT vs. Mono | 4 (836) | not serious | not serious ^b^ | not serious | serious ^c^ | **RR 0.95** (0.55 to 1.62) | **7 fewer per 1,000** (from 61 fewer to 84 more) | ⨁⨁⨁◯ MODERATE |
| Double vs. Mono | 1 (26) | not serious | not serious ^b^ | not serious | serious ^c^ | **-** | - | ⨁⨁⨁◯ MODERATE |
| **Neurological sequelae** | | | | | | | | |
| CS+ABT vs. Double | 1 (30) | not serious | not serious ^b^ | not serious | very serious ^c^ | **RR 1.93**  (0.37 to 10.01) | **116 fewer per 1,000** (from 79 fewer to 1000 more) | ⨁◯◯◯ VERY LOW |
| CS+ABT vs. Mono | 4 (777) | not serious | not serious ^b^ | not serious | serious ^c^ | **RR 0.95** (0.55 to 1.62) | **16 fewer per 1,000** (from 145 fewer to 199 more) | ⨁⨁⨁◯ MODERATE |
| Double vs. Mono | 1 (26) | not serious | not serious ^b^ | not serious | serious ^c^ | **-** | - | ⨁⨁⨁◯ MODERATE |
| **Any hearing loss** | | | | | | | | |
| CS+ABT vs. Double | 1 (30) | not serious | serious a | not serious | very serious ^c^ | **RR 1.81** (0.48 to 6.83) | **152 fewer per 1,000** (from 98 fewer to 1000 more) | ⨁◯◯◯ VERY LOW |
| CS+ABT vs. Mono | 4 (777) | not serious | not serious ^b^ | not serious | not serious | **RR 0.80** (0.55 to 1.14) | **38 fewer per 1,000** (from 87 fewer to 27 more) | ⨁⨁⨁◯ MODERATE |
| Double vs. Mono | 1 (26) | not serious | not serious ^b^ | not serious | serious ^c^ | **-** | - | ⨁⨁⨁◯ MODERATE |

Abbreviations: Mono, Mono antibiotic therapy; Double, Dual antibiotic therapy; CS, Corticosteroids; ABT, Antibiotic(s); CI, Confidence interval; RR, Risk ratio

Explanations

a. The study has high risk of bias according to RoB2.0 (Bhaumik, S & Begari, M 1998).

b. Low heterogeneity (I-square) across trials

c. Wide confidence intervals and few events.

**Table 10.2 Network estimates and GRADE quality assessment for primary outcomes**

| **Certainty assessment** | | | | | **Effect** | | **Certainty** |  |
| --- | --- | --- | --- | --- | --- | --- | --- | --- |
| **Treatment comparison** | **Risk of bias** | **Inconsistency** | **Indirectness** | **Imprecision** | **Relative (95% CI)** | **Absolute (95% CI)** |  |  |
| All-cause mortality | | | | | | | | |
| CS+ABT vs. Double | serious ^a^ | not serious ^b^ | not serious | very serious ^c^ | **RR 2.62** (0.30 to 22.77) | **3 fewer per 1,000** (from 23 fewer to 0 fewer) | ⨁◯◯◯ VERY LOW |  |
| CS+ABT vs. Mono | not serious | not serious ^b^ | not serious | serious ^c^ | **RR 0.65** (0.42 to 1.03) | **1 fewer per 1,000** (from 61 fewer to 0 fewer) | ⨁⨁⨁◯ MODERATE |  |
| Double vs. Mono | not serious | not serious ^b^ | not serious | very serious ^d^ | **RR 0.58** (0.01 to 49.59) | **1 fewer per 1,000** (from 50 fewer to 0 fewer) | ⨁⨁◯◯ LOW |  |
| Neurological sequelae | | | | | | | | |
| CS+ABT vs. Double | serious ^a^ | not serious ^b^ | not serious | serious ^c^ | **RR 0.67**  (0.14 to 3.17) | **1 fewer per 1,000** (from 3 fewer to 0 more) | ⨁⨁◯◯ LOW |  |
| CS+ABT vs. Mono | not serious | not serious ^b^ | not serious | serious ^c^ | **RR 0.75** (0.47 to 1.18) | **1 fewer per 1,000** (from 145 fewer to 0 fewer) | ⨁⨁⨁◯ MODERATE |  |
| Double vs. Mono | not serious | not serious ^b^ | not serious | serious ^c^ | **RR 0.50** (0.10 to 2.47) | **1 fewer per 1,000** (from 2 fewer to 0 fewer) | ⨁⨁⨁◯ MODERATE |  |
| Any hearing loss | | | | | | | | |
| CS+ABT vs. Double | serious ^a^ | not serious ^b^ | not serious | serious ^c^ | **RR 0.72**  (0.21 to 2.49) | **1 fewer per 1,000** (from 2 fewer to 0 fewer) | ⨁⨁◯◯ LOW |  |
| CS+ABT vs. Mono | not serious | not serious ^b^ | not serious | serious ^c^ | **RR 0.64** (0.55 to 1.62) | **1 fewer per 1,000** (from 1 fewer to 0 fewer) | ⨁⨁⨁◯ MODERATE |  |
| Double vs. Mono | not serious | not serious ^b^ | not serious | serious ^c^ | **RR 0.72** (0.21 to 2.49) | **1 fewer per 1,000** (from 2 fewer to 0 fewer) | ⨁⨁⨁◯ MODERATE |  |

Abbreviations: Mono, Mono antibiotic therapy; Double, Dual antibiotic therapy; CS, Corticosteroids; ABT, Antibiotic(s); CI, Confidence interval; RR, Risk ratio

Explanations

a. The study has high risk of bias according to RoB2.0 (Bhaumik, S & Begari, M 1998).

b. No inconsistency detected according global inconsistency test.

c. Confidence interval crosses null value.

d. Confidence interval across null value and wide confidence interval.

**Appendix 11**

**Comparison-adjusted funnel plot for each outcome from the network meta-analyses**

## eFigure 11.1 Comparison-adjusted funnel plot for the network of all-cause mortality in all comparisons.


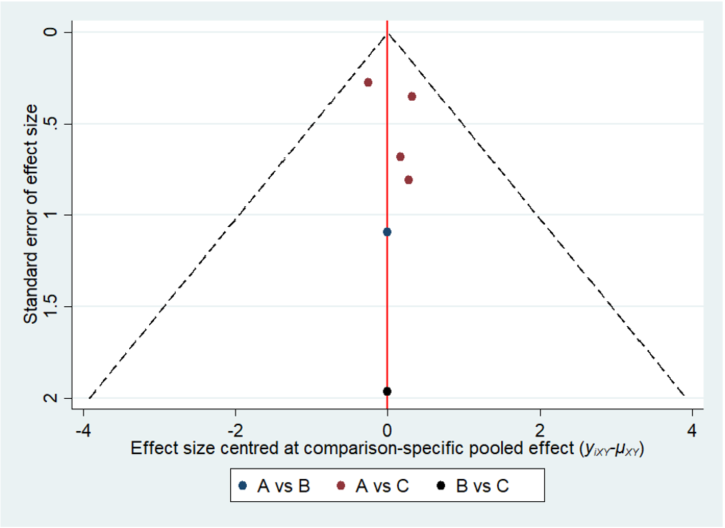


A, CS+ABT; B, Double; C, Mono

Abbreviations: Mono, Mono antibiotic therapy; Double, Dual antibiotic therapy; CS, Corticosteroids; ABT, Antibiotic(s)

## eFigure 11.2 Comparison-adjusted funnel plot for the network of neurological sequelae in all comparisons.


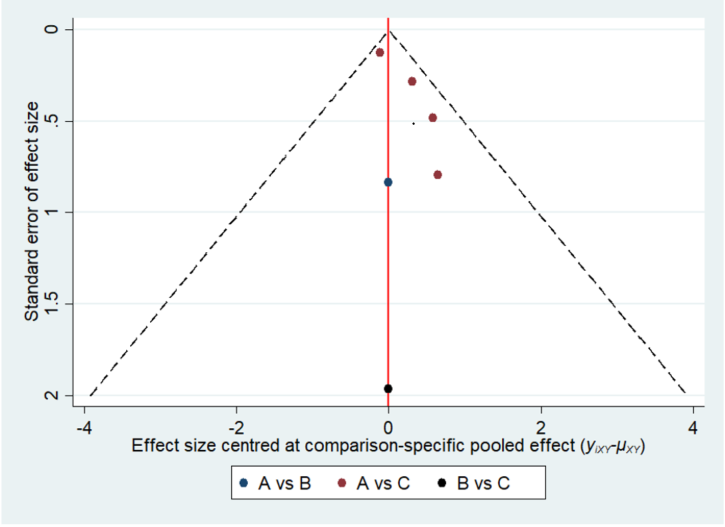


A, CS+ABT; B, Double; C, Mono

Abbreviations: Mono, Mono antibiotic therapy; Double, Dual antibiotic therapy; CS, Corticosteroids; ABT, Antibiotic(s)

## eFigure 11.3 Comparison-adjusted funnel plot for the network of any hearing loss in all comparisons.


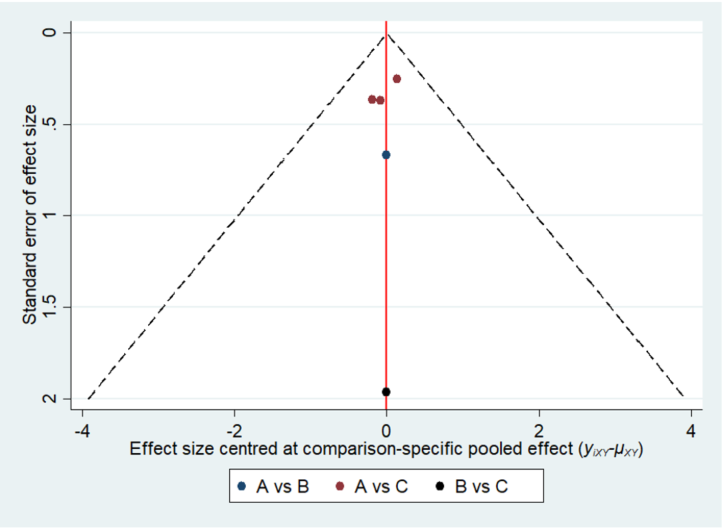


A, CS+ABT; B, Double; C, Mono

Abbreviations: Mono, Mono antibiotic therapy; Double, Dual antibiotic therapy; CS, Corticosteroids; ABT, Antibiotic(s)

# Appendix 12

## Trim-and-fill method for the pair-wise meta-analysis

## eFigure 12.1 Trim-and-fill method for the pair-wise meta-analysis of all-cause mortality


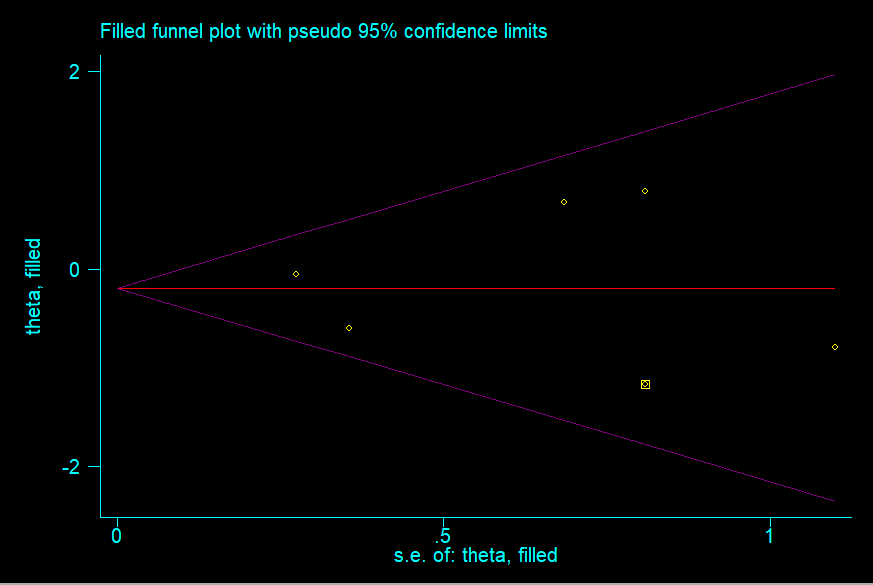


**Table 12.1 Results from the trim-and-fill method for publication bias in 6 studies for all-cause mortality**

| Filled meta-analysis | |  |  |  |  |  |
| --- | --- | --- | --- | --- | --- | --- |
| Model | **Summary logRR** | **95% CI** | | **Z value** | **p Value** | **Number of studies** |
| Fixed effect | -0.191 | -0.567 | 0.184 | -0.998 | 0.318 | 6 |
| Random effects | -0.189 | -0.666 | 0.288 | -0.776 | 0.438 |  |

RR, risk ratio

## eFigure 12.2 Trim-and-fill method for the pair-wise meta-analysis of neurological sequelae


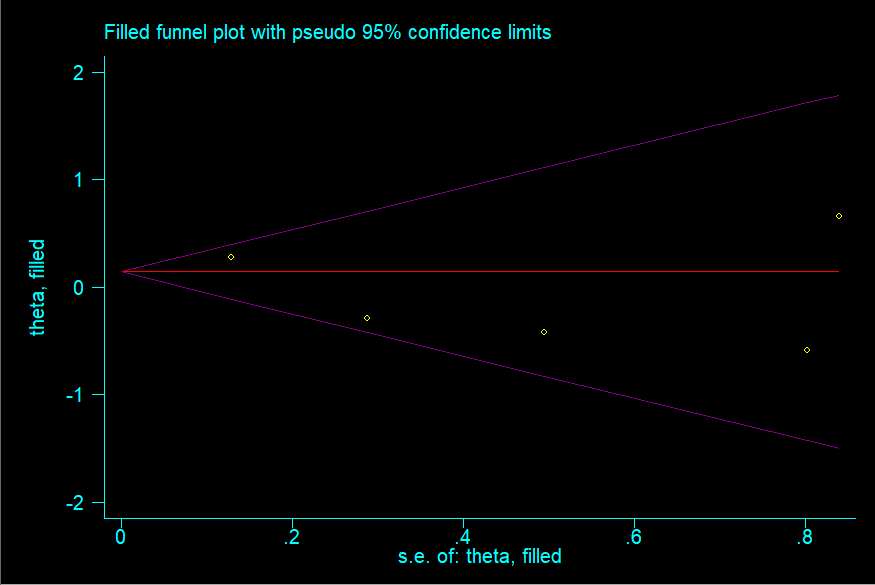


**12.2 Results from the trim-and-fill method for publication bias in 5 studies for neurological sequelae**

| Filled meta-analysis | |  |  |  |  |  |
| --- | --- | --- | --- | --- | --- | --- |
| Model | **Summary logRR** | **95% CI** | | **Z value** | **p Value** | **Number of studies** |
| Fixed effect | 0.142 | -0.078 | 0.363 | 1.264 | 0.206 | 5 |
| Random effects | 0.012 | -0.366 | 0.389 | 0.060 | 0.952 |  |

RR, risk ratio

## eFigure 12.3 Trim-and-fill method for the pair-wise meta-analysis of any hearing loss


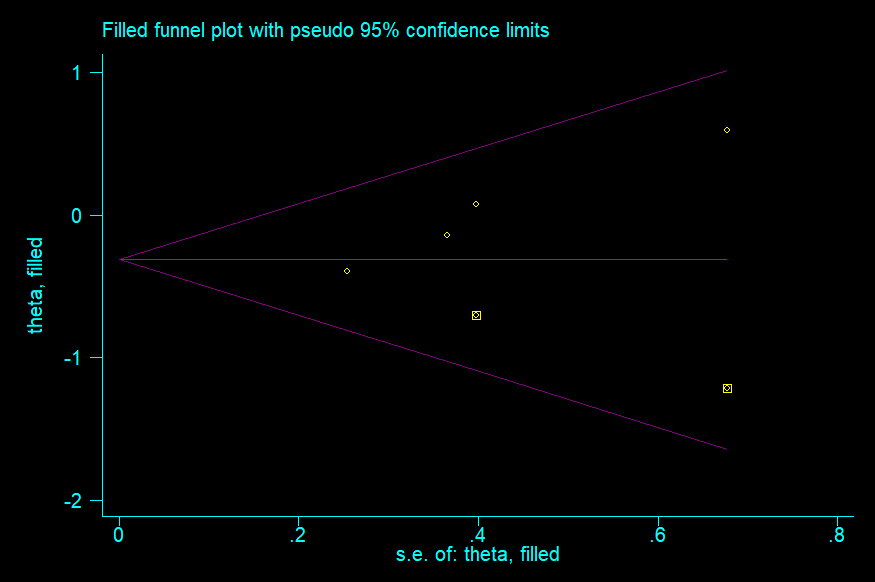


**Table 12.3 Results from the trim-and-fill method for publication bias in 6 studies for any hearing loss**

| Filled meta-analysis | |  |  |  |  |  |
| --- | --- | --- | --- | --- | --- | --- |
| Model | **Summary logRR** | **95% CI** | | **Z value** | **p Value** | **Number of studies** |
| Fixed effect | -0.313 | -0.623 | -0.003 | -1.980 | 0.048 | 6 |
| Random effects | -0.308 | -0.654 | 0.037 | -1.748 | 0.081 |  |

# Appendix 13 References

1. Bryan J, Rocha H, Silva H, Taveres A, Sande M, Scheld W. 1985. Comparison of ceftriaxone and ampicillin plus chloramphenicol for the therapy of acute bacterial meningitis. Antimicrobial agents and chemotherapy. 28(3):361-368.

2. Felipe Rucián A, García del Cerro G, Coll Usandizaga F, Tobeña Rué M, Martínez Gómez X, Moraga-Llop FA. 2011. Treatment of pneumococcal meningitis with dexamethasone. Anales de Pediatría 74:38-41.

3. Girgis N, Yassin M, Sanborn W, Burdick R, el-Ela H, Kent D, Sorensen K, Nabil I. 1972. Ampicillin compared with penicillin and chloramphenicol combined in the treatment of bacterial meningitis. Journal of tropical medicine and hygiene. 75(8):154-157.

4. Girgis NI, Abu el-Ella AH, Farid Z, Haberberger RL, Woody JN. 1988. Ceftriaxone alone compared to ampicillin and chloramphenicol in the treatment of bacterial meningitis. Chemotherapy 34 Suppl 1:16-20.

5. Girgis N, Farid Z, Mikhail I, Farrag I, Sultan Y, Kilpatrick M. 1989. Dexamethasone treatment for bacterial meningitis in children and adults. Pediatric infectious disease journal. 8(12):848-851.

6. Ivler D, Thrupp LD, Leedom JM, Wehrle PF, Portnoy B. 1963. AMPICILLIN IN THE TREATMENT OF ACUTE BACTERIAL MENINGITIS. Antimicrobial agents and chemotherapy 161:335-345.

7. Nathan N, Borel T, Djibo A, Evans D, Djibo S, Corty J, Guillerm M, Alberti K, Pinoges L, Guerin P, Legros D. 2005. Ceftriaxone as effective as long-acting chloramphenicol in short-course treatment of meningococcal meningitis during epidemics: a randomised non-inferiority study. Lancet (london, england). 366(9482):308-313. doi:10.1016/S0140-6736(05)66792-X.

8. Mathies AW, Leedom JM, Thrupp LD, Ivler D, Portnoy B, Wehrle PF. 1965. Ampicillin as a single drug in bacterial meningitis. The Journal of Pediatrics 67:1053.

9. Overturf GD, Steinberg EA, Underman AE, Wilkins J, Leedom JM, Mathies AW, Jr., Wehrle PF. 1977. Comparative trial of carbenicillin and ampicillin therapy for purulent meningitis. Antimicrob Agents Chemother 11:420-6.

10. Pécoul B, Varaine F, Keita M, Soga G, Djibo A, Soula G, Abdou A, Etienne J, Rey M. 1991. Long-acting chloramphenicol versus intravenous ampicillin for treatment of bacterial meningitis. The Lancet 338:862-866.

11. Pecoul B, Vaine F, Keita M, Soga G, Djibo A, Soula G. 1994. Chloramphenicol as an oily suspension for the treatment of bacterial meningitis. Annales de pediatrie. 41(4):205-209.

12. Thomas R, Le T, Bellissant E. 1996. The role of corticosteroid therapy on bacterial meningitis in adults. Medecine et maladies infectieuses. 26(Spec. Iss. Dec.):1119-1124. doi:10.1016/S0399-077X%2896%2980026-6.

13. Bademosi O, Falase AO, Jaiyesimi F, Bademosi A. 1976. Neuropsychiatric manifestations of infective endocarditis: a study of 95 patients at Ibadan, Nigeria. J Neurol Neurosurg Psychiatry 39:325-9.

14. Wali SS, et al. 1979. Single injection treatment of meningococcal meningitis. 2. Long-acting chloramphenicol. Transactions of the Royal Society of Tropical Medicine and Hygiene 73:698-702.

15. Buke A, Cavusoglu C, Karasulu E, Karakartal G. 2003. Does dexamethasone affect ceftriaxone penetration into cerebrospinal fluid in adult bacterial meningitis. International journal of antimicrobial agents. 21(5):452-456.

16. Chaudhary M, Shrivastava S, Sehgal R. 2008. Efficacy and safety study of fixed-dose combination of ceftriaxone-vancomycin injection in patients with various infections. Current drug safety. 3(1):82-85.

17. Fujii R. 1979. Results of a multicentric clinical study of mezlocillin in Japan (author's transl). Arzneimittel-forschung. 29(12a):2005-2008.

18. Hoepelman I, Rozenberg-Arska M, Verhoef J. 1988. COMPARISON OF ONCE DAILY CEFTRIAXONE WITH GENTAMICIN PLUS CEFUROXIME FOR TREATMENT OF SERIOUS BACTERIAL INFECTIONS. The Lancet 331:1305-1309.

19. Marra F, Reynolds R, Stiver G, Bryce E, Sleigh K, Frighetto L, MacDougall C, Jewesson P. 1998. Piperacillin/tazobactam versus imipenem: a double-blind, randomized formulary feasibility study at a major teaching hospital. Diagn Microbiol Infect Dis 31:355-68.

20. Erdem H, Kilic S, Sener B, Acikel C, Alp E, Karahocagil M, Yetkin F, Inan A, Kecik-Bosnak V, Gul HC, Tekin-Koruk S, Ceran N, Demirdal T, Yilmaz G, Ulu-Kilic A, Ceylan B, Dogan-Celik A, Nayman-Alpat S, Tekin R, Yalci A, Turhan V, Karaoglan I, Yilmaz H, Mete B, Batirel A, Ulcay A, Dayan S, Seza Inal A, Ahmed SS, Tufan ZK, Karakas A, Teker B, Namiduru M, Savasci U, Pappas G. 2013. Diagnosis of chronic brucellar meningitis and meningoencephalitis: the results of the Istanbul-2 study. Clinical Microbiology & Infection 19:E80-6.

21. Bennett IL, Finland, M, Hamburger, M, Kass, EH, Mark Lepper, M, Waisbren, BA. 1963. The Effectiveness of Hydrocortisone in the Management of Severe Infections: A Double-Blind Study. JAMA 183:462-465.

22. Fritz D, Brouwer M, Beek D. 2012. Dexamethasone and long-term survival in bacterial meningitis. Neurology. 79(22):2177-2179. doi:10.1212/WNL.0b013e31827595f7.

23. Mai NT, Hoa NT, Nga TV, Linh le D, Chau TT, Sinh DX, Phu NH, Chuong LV, Diep TS, Campbell J, Nghia HD, Minh TN, Chau NV, de Jong MD, Chinh NT, Hien TT, Farrar J, Schultsz C. 2008. Streptococcus suis meningitis in adults in Vietnam. Clin Infect Dis 46:659-67.

24. MacFarlane JT, Cleland PG, Attai ED, Greenwood BM. 1977. Failure of heparin to alter the outcome of pneumococcal meningitis. Br Med J 2:1522.

25. Scarborough M, Gordon S, Whitty C, French N, Njalale Y, Chitani A, Peto T, Lalloo D, Zijlstra E. 2008. Adult bacterial meningitis in malawi: a randomised controlled trail of steroid adjuvant therapy and a comparison of intravenous and intramuscular ceftriaxone. Journal of Infection 56:303.

26. Richards G, Elliott E, Shaddock E, Mushi D, Mzileni M, Ray R, Rulisa S, Seolwane F, Stacey S, Stoltz A, Venturas J, Schoeman H. 2013. A comparison of the pharmacokinetics of Aspen Ceftriaxone and Rocephin in community-acquired meningitis. South African medical journal. 103(12):906-909.

27. Bodilsen J, Dalager-Pedersen M, Schonheyder HC, Nielsen H. 2014. Stroke in community-acquired bacterial meningitis: a Danish population-based study. Int J Infect Dis 20:18-22.

28. Wang S-P, Zhao G-Y, Wang H. 2007. Cerebral spinal fluid replacement plus intrathecal administration in treatment of acute bacterial meningitis. Chinese journal of emergency medicine. 16(1):54-56.

29. Gupta A, Singh N. 1996. Dexamethasone in adults with bacterial meningitis. Journal of the Association of Physicians of India. 44(2):90-92.

30. Bhaumik S, Behari M. 1998. Role of dexamethasone as adjunctive therapy in acute bacterial meningitis in adults. Neurology india. 46(3):225-228.

31. de Gans J, van de Beek D. 2002. Dexamethasone in adults with bacterial meningitis. N Engl J Med 347:1549-56.

32. Gijwani D, Kumhar MR, Singh VB, Chadda VS, Soni PK, Nayak KC, Gupta BK. 2002. Dexamethasone therapy for bacterial meningitis in adults: a double blind placebo control study. Neurol India 50:63-7.

33. Nguyen TH, Tran TH, Thwaites G, Ly VC, Dinh XS, Ho Dang TN, Dang QT, Nguyen DP, Nguyen HP, To SD, Nguyen v V, Nguyen MD, Campbell J, Schultsz C, Parry C, Torok ME, White N, Nguyen TC, Tran TH, Stepniewska K, Farrar JJ. 2007. Dexamethasone in Vietnamese adolescents and adults with bacterial meningitis. N Engl J Med 357:2431-40.

34. Thomas R, Le Tulzo Y, Bouget J, Camus C, Michelet C, Le Corre P, Bellissant E. 1999. Trial of dexamethasone treatment for severe bacterial meningitis in adults. Adult Meningitis Steroid Group. Intensive Care Medicine 25:475-80.

35. Zavala I, Barrera E, Nava A. 1988. Ceftriaxone in the treatment of bacterial meningitis in adults. Chemotherapy. 34 Suppl 1(47-52.

36. Elyasi S, Khalili H, Dashti-Khavidaki S, Emadi-Koochak H. 2015. Conventional- versus high-dose vancomycin regimen in patients with acute bacterial meningitis: a randomized clinical trial. Expert Opin Pharmacother 16:297-304.

37. Narciso P, De M, Giannuzzi R. 1983. Ceftriaxon versus ampicillin therapy for purulent meningitis in adults. Drugs under experimental and clinical research. 9(10):717-719.

38. Schmutzhard E, Williams K, Vukmirovits G, Chmelik V, Pfausler B, Featherstone A. 1995. A randomised comparison of meropenem with cefotaxime or ceftriaxone for the treatment of bacterial meningitis in adults. Meropenem Meningitis Study Group. Journal of antimicrobial chemotherapy. 36 Suppl A(85-97.
