# Supplementary material for: Comparative efficacy of antibiotic(s) alone or in combination of corticosteroids in adults with acute bacterial meningitis: A systematic review and network meta-analysis
Source: PLoS One. 2020 May 29;15(5):e0232947. doi: 10.1371/journal.pone.0232947 (PMC7259733; doi:10.1371/journal.pone.0232947)
Supplement: Attachment — Submitted filename: Response_to_Reviewers_27032020_Clean.docx [file pone.0232947.s003.docx]

27 March 2020

Ahmed Negida, MD

Academic Editor

PLOS One

Email: ahmed.said.negida@gmail.com

Point-by-point responses to all comments are as follows:

**5. Review Comments to the Author**

**Reviewer #1: I have no comments to disclose to any author. The manuscript was technically and literally sound to me and I would like to thank the authors as well as the journal members for giving me the chance of reviewing such an article.**

Thank you very much. We really appreciate your valuable comments.

**Reviewer #2: The study highlights the importance of adjunct treatment of corticosteroid along with appropriate antibiotics in the treatment of bacterial meningitis to prevent the complications. The main age group of that study is the adult unlike most of the studies which focus on children.**

Thank you for your valuable comments. As highlighted by the reviewer, we had specifically focused on adult population since the treatment and causative pathogen of adult and paediatric meningitis are different and there is limited data in adult population which we believe will aid in future guidelines development.

**Reviewer #2: I think more tables and figures should be added eg; table for the demographic data for the included studies, the type of organism, and the treatment regimen, however, I see that meta-analysis will be a good add for the guidelines in the management of acute bacterial meningitis.**

**I also suggest if one of the biostatisticians reviews the statistical part of this meta-analysis and gives his feedback about the figure part.**

We have requested for our authors who are well versed in systematic reviews to help review the manuscript and data for accuracy. The figures have also been improved as suggested by the reviewer.

The table on key characteristics of included studies with information on the type of organism, the treatment regimen has been added in the manuscript. The revisions made are as followings.

**Original version**

None

**Revised version (line 610)**

**Table 1 Key characteristics of included studies**

| **Authors (year)** | **Country** | **Study design and size (N) (mean±SD)** | **Main pathogen (N)** | **Intervention** | **Comparator** | **Primary outcomes** |
| --- | --- | --- | --- | --- | --- | --- |
| CS+ABT vs. Double | | | | | | |
| Bhaumik, S. (1998)[27] | India | A single center, randomized clinical trial (N=30)  (28±17 vs. 34±19) | *N. meningitis* (n=6) *S. pneumoniae* (n=9) *Note:* No of isolates =15 | Dexamethasone (4 mg q 6 h 4 days then 4 mg tid on 5th day, bid on day 6th-7th) i.v. plus antibiotics ≥ 10 days (CS+PEN+CHLO)(n=14) *Note:* 13 patients were initially treated with C-pen (20 lac units IV q 4)+Chloramphenical (1 g IV q 6 h) Dexamethasone was started after 1^st^ dose of antibiotics | Antibiotics ≥ 10 days (PEN+CHLO) (n=16) *Note*: 14 patients were initially treated with C-pen (20 lac units IV q 4)+Chloramphenical (1 g IV q 6 h) | Death 1 (7.1) vs. 3 (18.8), p=0.60 Neurological sequelae: 3 (21.4) vs. 2 (12.5), p = 0.64 Audiological sequelae*: 4 (28.5) vs. 3 (18.75), p=1.00 *Note*: * Assessed by brainstem auditory evoked responses a/o pure tone auditory |
| CS+ABT vs. Mono | | | | | | |
| de Gans, J* (2002)[12] | Europe (The Netherlands, Belgium, Germany, Denmark, Austria) | A randomized, double-blind, multicenter trial (N=301)  (44±18 vs  46±20) | *S. pneumoniae* (n=108, 36%) *Neisseria meningitis* (n=97, 33% Others (n=29, 9.7%) Negative CSF culture (n=65, 21.3%)  *Note:* CSF culture was performed in 299 patients (155 vs. 144) | Dexamethasone sodium phosphate 10 mg q 6 h iv, 4 dys 15-20 mins before or with antibiotics (CS+PEN) (n=157)  *Note*: Most patients initially received amoxicillin 2 g iv q 4 h 7-10 dys 77% patients received amoxicillin and penicillin, 8% 3^rd^ generation cephalosporin, 8% amoxicillin or penicillin + cephalosporin | Placebo+antibiotic (PEN) (n=144)  *Note:* Most patients initially received amoxicillin 2 g iv q 4 h 7-10 dys | RR of death, 95%CI: 0.48 (0.24-0.96), p = 0.04  RR of an unfavorable outcome in intervention to comparator, 95%CI: 0.59 (0.37-0.394), p = 0.03  Focal neurologic abnormalities, 18/143 (13) vs. 24/119 (20); 95%CI: 0.62 (0.36-1.09), p=0.13  Hearing loss 13/143 (9) vs. 14/119 (12); 95%CI: 0.77 (0.38-1.58), p=0.54 |
| Gijwani, D (2002)[26] | India | A prospective placebo controlled, randomized double-blind study (N=40)  (28.25± 16.75 vs 32.25± 1.64) | *Pneumonococci* (n=8, 40%) *Staphylococci* (n=6, 30%) *Streptococci* (n=4, 20%) *H influenzae Streptococci* (n=4, 20%) *Meningococci* (n=2, 10%) E. Coli *Meningococci Streptococci* (n=2, 10%) | Dexamethasone (0.6 mg/kg/dy q 6 h first 4 days)+ceftriaxone 100 mg/kg/dy 14 days (CS+CEP) (n=20) *Note:* Dexamethasone was given at least 15 mins before ceftriaxone | Placebo+ ceftriaxone 100 mg/kg/dy 14 days (CEP) (n=20) | Mortality 2/18 (11.12) vs. 4/16 (25) Neurological sequelae at 90th day: 2/18 (11.12) vs. 4/16 (25) Hearing loss at 90th day: 7/18 (38.89) vs. 9/16 (56.25) |
| Nguyen, TH (2007)[30] | Vietnam | A randomized, double-blind, placebo-controlled trial (N=435)  Median age: 42 (15-89) vs. 41 (15-91) | *S. suis* (n=116, 52.3%) *S. pneumoniae* (n=55, 24.8%) *Streptococcus species* (n=18, 8.1%) *S. Aureus* (n=9, 4.1%) *N. meningitidis* (n=19, 8.6%) *H. influenzae* (n=7, 3.2%) *Klebsiella species* (n=10, 4.5%) *E. coli* (n=9, 4.1%) Other -ve bacteria (n=4, 1.8%) | Dexamethasone 0.4 mg/kg q 12 h, 4 dys 15 mins before antibiotics + ceftriaxone 2 g iv, q 12 h, 10-14 dys (CS+CEP) (n=217, 143*)  *Note:* Antibiotic treatment could be altered based on physician's discretion *Definite bacterial meningitis: If bacteria were detected in CSF or blood culture at the time of discharge or death | Placebo+ ceftriaxone 2 g iv, q 12 h, 10-14 dys (CEP) (n=218, 157*)  *Note:* Antibiotic treatment could be altered based on physician's discretion *Definite bacterial meningitis: If bacteria were detected in CSF or blood culture at the time of discharge or death | Death 1 month after randomization:18/217 (8.29) vs. 26/218 (11.93)  All patients: - RR of death at 1 months = 0.79 (0.45-1.39),ns - RR of death or disability at 6 months = 0.74 (0.47-1.17),ns  Definite BM: - RR of death at 1 month = 0.43 (0.20-0.94), p=0.03 - RR of death or disability at 6 months = 0.56 (0.32-0.98), p=0.03 |
| Thomas, R (1999)[25] | France and Switzerland | Multicenter, double-blind, randomized trial  (N=60)  (40±19 vs 50±19) | *S. pneumoniae* (n=31, 52%) *N. menigitidis* (n=18, 30%) Unknown (n=8, 13.3%) Others (n=3, 5%) | Aminopenicillin+ dexamethasone 10 mg qid 3 days (within 3 hours after aminopenicillin therapy initiation) (CS+PEN) (n=31)  *Note:* the first dose of dexamethasone was given within 3 hours after initiation of antibiotics | Aminopenicillin+placebo qid 3 days (within 3 hours after aminopenicillin therapy initiation) (PEN) (n=29) | The rate of patients cured without neurological sequelae at day 30: 23 (74) vs. 15 (52), p. 0.0711  Mild neurological sequelae at day 30: 2 (6.45%) vs. 4 (13.79%)  Severe neurological sequelae: 3 (9.68%) vs. 5 (17.24%) |
| Mono vs. Double | | | | | | |
| Zavala, I (1988)[28] | Mexico | An open, randomized comparative study (N=26)  28.6 (14-51) vs. 25.3 (16-52) | *S. pnuemoniae* (n=13)  *S. epidermidis* (n=3) *H. influenza* (n=2) *E. coli* (n=6) *S. typhi* (n=1) | Ceftriaxone i. v. 4 g OD (CEP) (n=13) *Note:* Dose decreased to 2 g when CSF became sterile | Ampicillin+ Chloramphenicol i.v. (PEN+CHLO) (n=13) *Note*: Ampicillin dose 200-400 mg/kg/dy, chloramphenicol dose 2-3 g/dy in 4 divided doses | The overall clinical and bacteriological cure rate (CSF sterile after 10 days): 100% (13/13) vs. 92% (12/13)* *Note:* *1 patient withdrew due to treatment failure |
| Others | | | | | | |
| Elyasi, S (2015)[34] | Iran | A randomized, open-labeled study (N=44)  (50.56± 18.22 vs 46.13± 17.11) | *S. pneumoniae* (n=25, 56.82%) *MRSA* (n=2, 4.54%) *S. epidermidis* (n=1, 2.27%) *E. faecalis* (n=1, 2.27%) *Note:* From 29/44 (65.9%) who had positive CSF culture | Vancomycin 15 mg/kg q 8 h (high dose)+Ceftriaxone 2g q 12h (n=22)  *Note*: All patients received 1st dose of antibiotics within 1 h of hospital admission. Most common regimen: Vancomycin plus ceftriaxone 2 g q 12 h (20 vs. 20) Target trough level = 15-20 mg/ml for ABM | Vancomycin 15 mg/kg q 12 h (conventional-dose)+ Ceftriaxone 2g q 12 h (n=22) *Note:* All patients received 1st dose of antibiotics within 1 h of hospital admission Target trough level = 15-20 mg/ml for ABM | GCS at 10th dy: 11 vs. 13, p = 0.02  CrCL at 10th day 102.14±44.24 vs 98.99±13.87 ml/min, p = 0.65 Time to normal WBC 3.22±3.11 vs 6.00±2.45, p=0.03 Time to afebrile 3.35±1.23 vs 6.11±2.00, p=0.02 Duration of hospitalization day: 12.85±5.47 vs 10.10±2.45, p = 0.04 |
| Narciso, P (1983)[35] | Italy | A single-center, randomized controlled trial (N=10)  (48 vs. 44) | *N. meningitidis* (n=3) E. coli (n=1) *D. pneumoniae* (n=3) *Note:* Negative culture in 3 patients | Ceftriaxone i.v. q 12 h 100-80 mg/kg/day in 4 cases and 45 mg/kg in 1 case (CEP) (n=5) | Ampicillin (110 mg/kg) divided in 3 slow iv q 8 hr (PEN) (n=5) | All patient recovered completely |
| Schmutzhard, E (1995)[36] | Hungary, the Czech Republic, Portugal, France, Spain, Austria (from 15 centres) | Two prospective randomized controlled studies (N=56)  Median 46 (17-76) vs. 31 (13-71) | *H. influenzae* 7.1% (n=1) *N. menigitidis* 39.3% (n=18) *S. pneumoniae* 78.6% (n=31) Others 3.57% (n=2) | Meropenem 40mg/kg q 8 h, up to a maximum of 6g/dy, 7-14 dys¶ (MPN) (n=28)  *Note*: Dexamethasone was administered to 39 patients (meropenem 19, cephalosporins 20) | Cephalosporins, 7-14 dys¶ (n=28): Cefotaxime (n=17) Ceftriaxone (CEP) (n=11)  *Note*: Dexamethasone was administered to 39 patients (meropenem 19, cephalosporins 20) | Clinical cured: Meropenem vs. Cephalosporins: 23/23 (100%) vs. 17/22 (77%)  Neurological sequelae: Meropenem 3 (10.7%) vs. Cephalosporins 4 (14.3%); 4 receiving cefotaxime  Hearing impairment: 11 (39.3%) vs. 9 (32%)  Death: Meropenem 3 vs. 1 Cephalosporins |

**Reviewer #3: Line 108: Sentence, "comprehensively examine the relative efficacy between corticosteroid" should be "......relative efficacy of corticosteroid"**

**Line 110: It is no appropriate to say synthesizing results". You can say "Processing or analyzing results..." instead**

**Response #3.1**

Thank you for your comment. The sentences have been revised accordingly as follows.

**Original version (lines 107-111)**

**Lines 107-111**

Therefore, this systematic review and network meta-analysis (NMA) was performed to comprehensively examine the relative efficacy between corticosteroids in combination with antibiotic treatment, dual antibiotic, and mono antibiotic therapies for both direct and indirect comparison in treating adult bacterial meningitis by synthesizing results from randomized controlled trials which are considered to be on the top level in the hierarchy of evidences.

**Revised version (lines 113-117)**

**Lines 113-117**

Therefore, this systematic review and network meta-analysis (NMA) was performed to comprehensively examine the relative efficacy of corticosteroids in combination with antibiotic treatment, dual antibiotic, and mono antibiotic therapies for both direct and indirect comparison in treating adult bacterial meningitis by analyzing results from randomized controlled trials which are considered to be on the top level in the hierarchy of evidences.

**Reviewer #3 Line 417-418: Sentence, "To the best of our knowledge .........focusing in..." should be '...focusing on.."**

**Lines 417-419**

To the best of our knowledge this is the first systematic review and network meta-analysis comparing efficacy between different pharmacological treatments focusing in adult bacterial meningitis.

**Revised version (Lines 430-432)**

To the best of our knowledge this is the first systematic review and network meta-analysis comparing efficacy between different pharmacological treatments focusing on adult bacterial meningitis.

**Reviewer #4: The authors performed a systematic review and network meta-analysis to evaluate the benefit of adding corticosteroids to antibiotics. The search and analysis methods are proper (despite the old search date). I have some comments:**

*** Title: After reading the manuscript, the title should be changed to "Antibiotics alone or in combination with corticosteroids in adults with acute bacterial meningitis. This is because these were the only two modalities investigated in this study. Also, the title should mention this is a network meta-analysis. The short title needs to be shorter.**

**Response #4.1**

Thank you for your suggestion. The title has been revised accordingly from **“Comparative efficacy of different pharmacotherapies for adults with acute bacterial meningitis: a systematic review and GRADE analysis”** to be **“Comparative efficacy of antibiotic(s) alone or in combination of corticosteroids in adults with acute bacterial meningitis: A systematic review and network meta-analysis”**. The short title has also been revised from **“Comparative efficacy of different pharmacotherapies for adult bacterial meningitis”** to be “**Comparative efficacy in adult bacterial meningitis treatments”.** The revisions have been done in the manuscript as follows:

**Original version (Line 1-3)**

Comparative efficacy of different pharmacotherapies for adults with acute bacterial meningitis: a systematic review and GRADE analysis

**Revised version (Line 1-3)**

Comparative efficacy of antibiotic(s) alone or in combination of corticosteroids in adults with acute bacterial meningitis: a systematic review and network meta-analysis

**Reviewer #4: Abstract- Why did you phrase hearing loss as more important than neurological complications? Please explain.**

**Response #4.2**

Thank you for your comment. We did not phrase hearing loss as more important than neurological complications but according to our network meta-analysis results corticosteroids in combination with antibiotic therapy was significantly more effective in reducing the risk of any hearing loss compared to mono antibiotic therapy (RR 0.64; 95%CI, 0.45 to 0.91, 4 RCTs, moderate certainty of evidence) whereas numerical lower risk of mortality and neurological complications were observed for adjunctive corticosteroids in combination with antibiotic therapy versus mono antibiotic therapy but the numbers were non-significant.

**Reviewer #4: The search date is two years old. This needs to be updated.**

**Response #4.3**

Thank you for the comment. We have performed an updated search from 2018-2020 and identified no new studies to be included in our analyses. We included randomized controlled trials in adult with acute bacterial meningitis and excluded studies in paediatrics, mycobacterium, aseptic, viral or nosocomial meningitis as well as HIVs and immunocompromised patients. We have also mentioned regarding an updated search in the PRISMA flow chart (Figure 1), Appendix 1, abstract section (lines 54-56), and Search Strategy and study selection (lines 135-136) as follows:

**Original version (lines 53-54)**

None

**Revised version (lines 54-56)**

**Lines 54-56**

An updated search from 9 February to 9 March 2020 was performed, and no new studies met the inclusion criteria.

**Lines 135-136**

Additionally, we performed an updated search from 2018-2020 up to 9 March 2020 and identified no new studies to be included in our analyses.

**Reviewer #4: - The results should indicate the presented findings are in comparison with mono or double antibiotics!**

**Response #4.4**

This may not have been clear in our initial submission. We have revised the statement on comparison with mono or double antibiotics had already been indicated previously as below (lines 66-68):

“Network meta-analysis suggests that corticosteroids in combination with antibiotic therapy was more effective in reducing the risk of any hearing loss compared to mono antibiotic therapy (RR 0.64; 95%CI, 0.45 to 0.91, 4 RCTs, moderate certainty of evidence)” (lines 66-68).

**Reviewer #4** **The abstract should present briefly the results of pairwise meta-analysis for the three main outcomes, followed by NMA.**

**Response #4.5**

Thank you for the comment. The results of pairwise meta-analysis were presented in the abstract as follows:

**Original version**

None

**Revised version (line 65)**

No significant difference between treatment was noted in meta-analysis.

**Reviewer #4 Also, the effect estimates and 95% for all primary outcomes should be reported!**

**Response #4.6**

Thank you for the comment. The effect estimates and 95% for all primary outcomes have been reported in the abstract as follows:

**Original version (lines 65-67)**

Network meta-analysis suggests that corticosteroids in combination with antibiotic therapy was more effective in reducing the risk of any hearing loss compared to mono antibiotic therapy (RR 0.64; 95%CI, 0.45 to 0.91, 4 RCTs, moderate certainty of evidence).

**Revised version (lines 66-68, 68-72)**

Network meta-analysis suggests that corticosteroids in combination with antibiotic therapy was more effective in reducing the risk of any hearing loss compared to mono antibiotic therapy (RR 0.64; 95%CI, 0.45 to 0.91, 4 RCTs, moderate certainty of evidence).

Numerical lower risk of mortality and neurological complications was also shown for adjunctive corticosteroids in combination with antibiotic therapy versus mono antibiotic therapy (RR 0.65; 95%CI, 0.42 to 1.02, 6 RCTs, moderate certainty of evidence; RR 0.75; 95%CI, 0.47 to 1.18, 6 RCTs, moderate certainty of evidence).

**Reviewer #4- Lines 66-68: This outcome is not significant in the results and should be identified here as such. This is benefit of adding numbers!**

**Response #4.7**

Thank you for the comment. The effect estimates and 95% for all primary outcomes have been reported in the abstract as indicated in the previous response (Response #4.6):

**Reviewer #4-**- **In the conclusion, you only mention hearing loss outcome! It is the only significant outcome, but other outcomes should not be ignored, just because they are insignificant.**

**Response #4.8**

Thank you for your comment. We have reported other outcomes (all-cause mortality and neurological complications) and indicated the effect estimates and 95% CI of them in line 68-72 as follows.

**Original version (lines 66-68)**

Numerical lower risk of mortality and neurological complications was also shown for adjunctive corticosteroids in combination with antibiotic therapy versus mono antibiotic therapy.

**Revised version (lines 68-72)**

Numerical lower risk of mortality and neurological complications was also shown for adjunctive corticosteroids in combination with antibiotic therapy versus mono antibiotic therapy (RR 0.65; 95%CI, 0.42 to 1.02, 6 RCTs, moderate certainty of evidence; RR 0.75; 95%CI, 0.47 to 1.18, 6 RCTs, moderate certainty of evidence).

**Reviewer #4-** **Introduction: well-written and provides a rationale that sets the stage for the network meta-analysis. I would just recommend because most readers are not yet familiar with NMA to add some information about it before the objective.**

**Response #4.9**

Thank you for this useful suggestion. We have added the information about NMA before the objective accordingly as follows:

**Original version**

None

**Revised version (lines 108-112)**

With limited head-to-head studies, application of network meta-analysis (NMA), an analytical approach that does not only include direct comparisons, but also indirect comparisons in addition to traditional meta-analysis that assess the treatment effects based on pair-wise head to head direct comparisons [14, 15] would be useful to assist in decisions.

**Reviewer #4-The methods are well-written and is detailed enough to allow replication of the analysis. As mentioned above, the search date is too old (two years) and will be more if published. Therefore, the search must be updated!**

**Response #4.10**

Thank you for this valuable comment. We have performed an updated search and revised the manuscript (lines 54-56 in abstract section and 132-133) and the PRISMA flow chart (Figure 1) accordingly as follows:

**Original version**

None

**Revised version (lines 135-136)**

Additionally, we performed an updated search from 2018-2020 up to 9 March 2020 and identified no new studies to be included in our analyses.

**Reviewer #4-PubMed is a search engine that also searches Medline. So, you mentioned you searched Medline and PubMed. So, what search engine you used for Medline?**

**Response #4.11**

Thank you for your comment. The search engine we used for Medline was Ovid. We have put “Ovid” before Medline to clarify this in the manuscript accordingly as below.

**Original version (lines 126-127)**

“We searched in nine relevant databases including CINAHL Plus, Cochrane Library, EMBASE, Global Health, Medline, PubMed, SciELO, Science Direct and Scopus..”

**Revised version (lines 131-132)**

“We searched in nine relevant databases including CINAHL Plus, Cochrane Library, EMBASE, Global Health, Ovid Medline, PubMed, SciELO, Science Direct and Scopus..”

**Reviewer #4-** **Line 130: "Will be" is the way of writing protocols!**

**Response #4.12**

Thank you for your comment. We have revised “will be” to be “would be” in the manuscript as follows

**Original version (lines 130-131)**

“Articles will be included if they were randomized controlled trials (RCTs) evaluating on pharmacological treatment and clinical outcomes in adult bacterial meningitis with at least one….”

**Revised version (lines 136-138)**

“Articles would be included if they were randomized controlled trials (RCTs) evaluating on pharmacological treatment and clinical outcomes in adult bacterial meningitis with at least one…”

**Reviewer #4-** **Lines 137-138: Any response from these authors?**

**Response #4.13**

No response was received and we have added the sentence in the manuscript to clarify accordingly as below.

**Original version**

None

**Revised version (lines 144)**

However, there was no response received.

**Reviewer #4-** **No information on how screening was done are presented!**

**Response #4.14**

Thank you for the comment. The information about screening has already been mentioned in the method section (lines 144-147) as follows.

**Original version**

None

**Revised version (lines 144-147)**

Two reviewers (AR and H-LS) independently screened and selected articles by title and abstract. The full text eligibility was also assessed independently. Any discrepancies were discussed and resolved with consensus with other authors (PP, K-GC, B-HG, T-MK, SS, SWHL, and L-HL) to reach agreement.

**Reviewer #4-Outcomes are well-defined. The same is true for quality assessment and data analysis. Have you attempted P score ranking?**

**Response #4.15**

Thank you for your comment. Similar to SUCRA, P-score ranking provides a ranking of all treatments according to the point estimates but only takes standard errors into account which does not seem to provide additional advantages to SUCRA ranking. Therefore, we did not perform P-score ranking since there is limited additional value and information that could be added in our analysis.

**Reviewer #4-** **Lines 231-233: It is not clear which outcome is superior to others!**

**Response #4.16**

Thank you for your comment. We have revised the sentence to read as follows (Lines 235-238)

“For all-cause mortality, corticosteroids in combination with antibiotic vs. mono antibiotic therapy was ranked the most effective, followed by corticosteroids in combination with antibiotic and finally dual antibiotic therapy.”

**Reviewer #4-The authors should insert the numbers of effect estimate and significance measures to the NMA results section.**

**Response #4.17**

Thank you for the comment. The numbers of effect estimate and significance measures together with corresponding 95% confidence intervals (95% CIs) have been inserted accordingly as follows.

**Original version**

**Lines 262-263**

The NMA results involving 833 patients found no significant differences observed for any treatment comparison (Figure 3B) (S2 Appendix 8, eFigure 8.2).

**Revised version**

**Lines 266-271**

The NMA results involving 833 patients found no significant differences observed for any treatment comparison (adjunctive corticosteroids in combination with antibiotic therapy vs. mono antibiotic therapy; RR 0.75; 95%CI, 0.47 to 1.18 and dual antibiotic therapy vs. mono antibiotic therapy; RR 0.50; 95%CI, 0.10 to 2.47, 6 RCTs, moderate certainty of evidence; dual antibiotic therapy vs. adjunctive corticosteroids in combination with antibiotic therapy; RR 0.67; 95%CI, 0.14 to 3.17, 6 RCTs, low certainty of evidence) (Table 2) (Figure 3B) (S2 Appendix 8, eFigure 8.2).

**Reviewer #4-** **As per my understanding, all pairwise meta-analyses were non-significant, so did all NMAs, except for hearing loss. In terms of SCURA ranking, Cs plus ABs ranked first in one outcome, while double antibiotics ranked first in the two other primary outcomes. Although the analysis methods seem proper, it is hard to derive a consistent clinical implication that recommends adding Cs to ABs based on these outcomes. My advice is to repeat the search and try to include the more recent studies to see how the final analysis would turn out.**

**Response #4.18**

Thank you for the comment. We agree that this was confusing and as suggested by the esteemed reviewer, we have conducted additional search but no studies met the inclusion criteria and thus were included.

Based upon the SUCRA ranking, the use of Cs plus Abs ranked first for all-cause mortality was probably because of the number of studies that were used in the analyses (8 studies versus 6 studies for neurological complications and 4 studies for any hearing loss). This is shown in the wide estimates obtained for the latter estimates. Nevertheless, as suggested by the reviewer, we have included this as a limitation of our study in the discussion (lines 395-402).

The revisions in the discussion section are as follows:

**Original version**

None

**Revised version**

**Lines 395-402**

In the treatment relative ranking, corticosteroids in combination with antibiotic ranked first for all-cause mortality outcome while dual antibiotic therapy ranked first for neurological complication and any hearing loss. This is due to the lack of studies which had reported the latter outcomes (8 studies for all-cause mortality vs. 6 studies for neurological sequelae and 4 studies for any hearing loss). As such, the evidence from SUCRA ranking is of low certainty and therefore could be unreliable since the magnitudes of difference between effect estimates are not accounted [33]. Under such we suggest that the interpretation of results should be on NMA findings and GRADE analysis (Table 2).

**Reviewer #4-** - **Lines 371-374: The authors keep repeating the fact of lower numerical RR despite non-significance. In my opinion, it is just non-significant. Again, interpreting a solid consistent clinical implication based on the above-mentioned results is difficult.**

**Response #4.19**

Thank you for your comment. The sentences mentioned have been removed from the manuscript as follows:

**Original version (lines 371-374)**

In term of all-cause mortality and neurological complications, corticosteroids in combination with antibiotic therapy showed numerical lower risk of death and neurologic sequelae compared to mono antibiotic therapy despite non-significant finding.

**Revised version**

Removed

**Reviewer #4-** The discussion should be better structured into shorter, more focused paragraphs. Following the limitations, add your recommendations for future practice and clinical research.

**Response #4.20**

Thank you for this valuable comment. We have revised the discussion part as well as added recommendations for future practice and clinical research accordingly as follows:

**Original version (lines 371-374)**

In term of all-cause mortality and neurological complications, corticosteroids in combination with antibiotic therapy showed numerical lower risk of death and neurologic sequelae compared to mono antibiotic therapy despite non-significant finding.

**Revised version**

Removed

**Original version (lines 420-422)**

Exhaustive quality assessment, data extraction and analyses were done with confirmation by at least among two reviewers in order to make the best use of limited evidences in adult population with ABM.

**Revised version (lines 433-435)**

Exhaustive quality assessment, data extraction and analyses were done with confirmation by at least among two reviewers in order to make the best use of limited evidences.

**Original version (lines 430-431)**

Attempts to contact the corresponding authors were also made for further clarification.

**Revised version (lines 455-456)**

Removed

**Original version**

None

**Revised version (lines 463-467)**

The likely benefit is anticipated if corticosteroids is given before or together with antibiotic treatment. Timely and sufficient treatment are essential to the disease outcomes. In settings with high prevalence of resistant pneumococcal strains (to penicillin and cephalosporins), dual antibiotic therapy (vancomycin in combination with a cephalosporin) should be opted rather than mono antibiotic therapy.

**Original version**

None

**Revised version (lines 468-470)**

Future research to provide a better understanding of acute bacterial meningitis mechanism and corticosteroids effects on the disease would be helpful in identifying optimal treatment strategies.

We thank the reviewers and editor for their invaluable time to improve our manuscript and look forward to contributing to your esteemed journal

Yours sincerely,

Ajaree Rayanakorn

Ajaree Rayanakorn, B.Pharm, MPH (Tropical Health).

Ph.D Candidate, Jeffrey Cheah School of Medicine and Health Sciences

Monash University Malaysia

Email: [ajaree.rayanakorn@monash.edu](mailto:ajaree.rayanakorn@monash.edu),

Tel: 60 3 5516 1241
